# Mitogenomic phylogenetic analyses provide novel insights into the taxonomic problems of several hesperiid taxa (Lepidoptera: Hesperiidae)

**DOI:** 10.1038/s41598-023-34608-8

**Published:** 2023-05-16

**Authors:** Lijuan Zhu, Yuke Han, Yongxiang Hou, Zhenfu Huang, Min Wang, Hideyuki Chiba, Liusheng Chen, Xiaoling Fan

**Affiliations:** 1 Department of Entomology, College of Agriculture, South China Agricultural University, Guangzhou, 510642 China; 2 Guangdong Academy of Forestry, Guangzhou, 510520 China; 3 Guangdong Southern Newspaper Media Group Co., Ltd, Guangzhou, 510601 China; 4 School of Life Science and Engineering, Southwest University of Science and Technology, Mianyang, 621010 China; 5 B. P. Bishop Museum, Honolulu, HI 96817-0916 USA

**Keywords:** Molecular evolution, Phylogenetics, Taxonomy

## Abstract

Here, we present new molecular and morphological evidence that contributes towards clarifying the phylogenetic relations within the family Hesperiidae, and overcomes taxonomic problems regarding this family. First, nine new complete mitogenomes, comprising seven newly sequenced species and two samples of previously sequenced species collected from different localities, were obtained and assembled to analyze characteristics. The length of the mitogenomes ranges from 15,284 to 15,853 bp and encodes 13 protein-coding genes, two ribosomal RNA (rRNA) genes, 22 transfer RNA (tRNA) genes, and a control region. Two model-based methods (maximum likelihood and Bayesian inference) were used to infer the phylogenetic relationships. Based on the mitogenomic phylogenetic analyses and morphological evidence, we claim that the lineage that comprises two Asian genera, *Apostictopterus* Leech and *Barca* de Nicéville, should be a tribe Barcini stat. nov. of the subfamily Trapezitinae, *Pseudocoladenia dea* (Leech, 1894), *P. festa* (Evans, 1949), and *Abraximorpha esta* Evans, 1949 are considered distinct species. Finally, we suggest that *Lotongus saralus chinensis* Evans, 1932 should belong to the genus *Acerbas* de Nicéville, 1895, namely *Acerbas saralus chinensis* (Evans, 1932) comb. nov..

## Introduction

Advances in molecular techniques accelerate our understanding of biological diversity and phylogenetic relationships of taxa. Taxonomic research now largely relies on molecular phylogeny. However, there is still opportunity for taxonomic decisions to determine the taxonomic rank that should be assigned to a particular taxon.

Hesperiidae is the third-largest butterfly family, containing approximately 600 genera and 4300 species^1–3^. Recent higher-level molecular phylogenetic studies of the family have unveiled taxonomic inconsistencies that have attracted research attention^1,2,4–9^. The taxonomic interpretation is under more debate than the differences in phylogeny. Warren et al.^10^ proposed seven subfamilies, which is still followed by current reserach^4,6^. However, Zhang et al. divided the family into additional subfamilies including Katreinae, Chamundinae, and Barcinae^8^. The treatment of Pyrrhopyginae as a subfamily was followed in other literature^10,11^. However, these taxonomic conflicts remain unsettled.

The subfamily level, Pyrginae sensu lato and Barcinae are the focus of current study. Morphologically, the subfamily Pyrginae had been speculated as a polyphyletic group^12–14^, However, taxonomists have not attempted to divide the subfamily into appropriate groups until this is supported by molecular phylogenetic studies using several gene markers^1,4,15^. Based on three genes and 49 morphological characteristics, Warren et al.^16^ treated Pyrginae as a monophyletic group and the finding was extensively supported by current research^4,6,7,17^. Meanwhile, Li et al.^7^ divided the subfamily Pyrginae sensu lato (i.e., sensu Warren et al., 2009) into three subfamilies, namely Tagiadinae, Pyrrhopyginae, and Pyrginae based on their relative divergence time compared to other subfamilies^7^.

Two Asian genera, *Apostictopterus* and *Barca*, have been traditionally classified in the Heteropterus genus group of the subfamily Hesperiinae^9,18,19^ or in a distinct subfamily Heteropterinae^1,20^ based on morphology. Analysis of mitogenomic data, however, suggested that both *Barca* and *Apostictopterus* are not within the Heteropterin genera and should tentatively be assigned to Hesperiinae, owing to the absence of the subfamily Trapezitinae^17^ in the phylogeny. Phylogenetic analyses based on whole-genome data have demonstrated showed that the clade of these two genera is a sister to that of the Trapezitinae^8^. Based on the result, Zhang et al. proposed that *Barca* and *Apostictopterus* should be classified as members of a new subfamily Barcinae^8^. In our study, we aimed to challenge this taxonomic assumption.

Recent studies show that the mitochondrial genome can provide good phylogenetic signals for understanding taxonomic systematics^8,17,21–23^. Yoshizawa et al. used mitochondrial genomic data to construct phylogenetic trees and explore mitochondrial evolution problems in Psocodea, providing strong support for the Prionoglarididae family because its monophyletics were inconsistent in previous morphological and molecular studies^23^. To better understand the phylogenetic relationships among the subfamilies and genera of Hesperiidae, we sequenced and assembled the mitogenomes of 13 samples representing 13 species/subspecies, including four taxa (*Pseudocoladenia dea*, *Pseudocoladenia festa*, *Abraximorpha esta*, and *Lotongus saralus chinensis*) whose taxonomic statuses remain unsettled. In a previous study, Huang & Xue elevated three subspecies of *Pseudocoladenia dan*, *Pesudocoladenia dan dea* (type locality: Pu-tsu-fong, Sichuan), *P. dan festa* (type locality: Naga Hill, India), and *P. dan fauta* (type locality: Gangtok, Sikkim, India), to species level based on morphological characteristics^24^. Further, *Lotongus saralus* de Nicéville, 1889, a skipper from the Oriental region, was transferred to *Acerbas* based only on genome data^2,9^. Using publicly available mitogenomic sequences of 45 skipper species in GenBank (Supplementary Table S1) as well as our 13 mitogenomes, we reconstructed phylogenetic trees of the 58 skipper species using both maximum likelihood (ML) and Bayesian inference (BI) methods. Finally, based on the molecular results and morphological analysis, we aimed to clarify the monophyly of Pyrginae, the taxonomic positions of *Apostictopterus* and *Barca*, and the taxonomic status of the four taxa mentioned above.

## Results and discussion

### Mitogenome features

**Figure 1:**
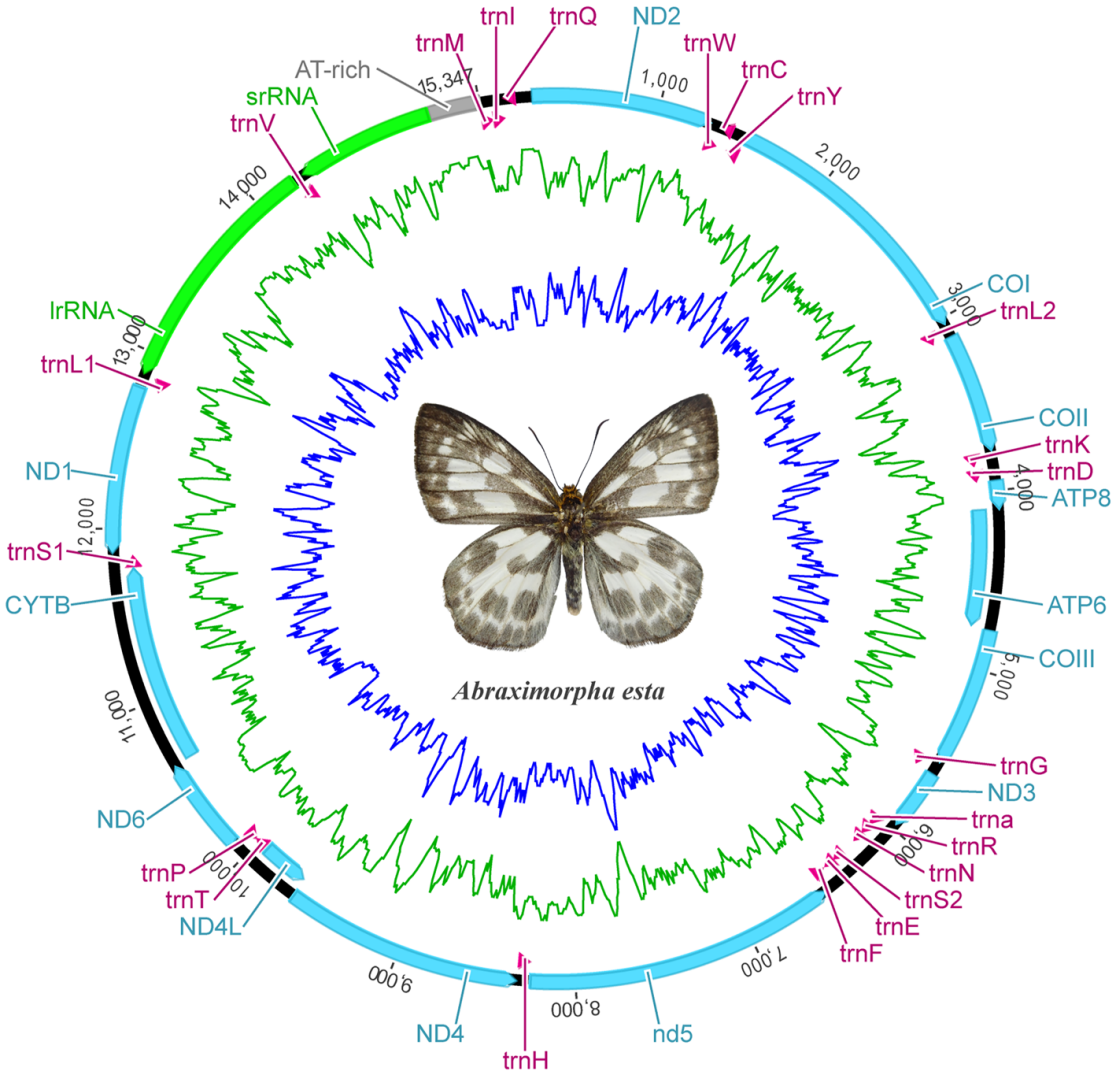
A circular map of the *Abraximorpha esta* mitochondrial genome.

Nine new mitogenomes were assembled and annotated. Their circular maps were similar, thus only the map of *Abraximorpha esta* is shown (Fig. 1, Supplementary Fig. S1). The length of the mitogenomes ranged from the shortest, 15,284 bp (*Coladenia maeniata*) to the longest, 15,853 bp (*Lotongus saralus chinensis* and *Trapezites iacchus*). Each mitogenome was typically composed of 13 PCGs, 22 tRNA genes, 2 rRNA genes, and one major non-coding AT-rich region. Twelve of the 13 PCGs started with three typical start codon types (ATA, ATG, and ATT) in skippers, however one, COI, began with CGA, which is common across the order Lepidoptera. The PCGs terminated with stop codons TAA or TAG or an incomplete stop codon T–; incomplete stop codons always appear in lepidopteran mitogenomic PCGs. All start and stop codons are listed in Supplementary Table S2.

**Figure 2:**
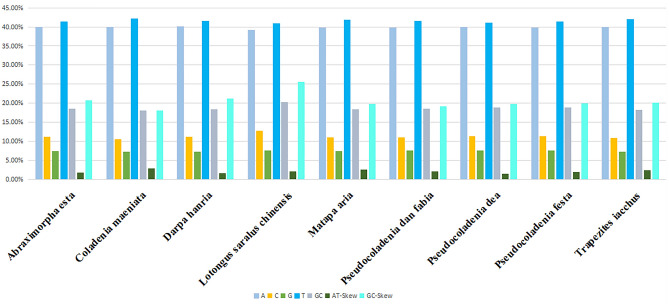
Analysis of base composition and skewness of nine mitochondrial genomes.

The nucleotide proportion, GC content, AT-skew, and GC-skew of the nine sequences were comprehensively calculated and analyzed (Fig. 2). The fluctuation in adenine (A) content in butterflies is generally small^25^, and the variation range of the nine sequences base A was ± 0.01. Among the nine sequences, *Lotongus saralus chinensis* had the highest GC-skew (25.63%), whereas *Pseudocoladenia dan fabia* had the lowest GC-skew (19.25%). The mean GC-skew was 20.88%, indicating that cytosine was present more frequently in genes than guanine. We analyzed the characteristics of the PCG nucleotide sequences (Table 1). Among the 13 PCGs, the COI gene had the highest number of invariant sites and the lowest percentage of variable sites. According to the percentage of variable sites, ND6 has the highest mutation rate, and COI has the smallest mutation rate.

**Table 1:** Analysis of nucleotide variation sites of protein-coding genes.

	Invariant sites	Variable sites	Parsimony-informative sites	Singleton variable sites	Percentage of variable sites	Total sites
*ATP6*	276	402	314	88	0.575	699
*ATP8*	56	118	105	13	0.656	180
*COI*	890	650	551	96	0.419	1551
*COII*	360	325	263	62	0.466	697
*COIII*	382	407	347	60	0.499	816
*CYTB*	555	603	521	82	0.517	1167
*ND1*	400	555	432	122	0.572	970
*ND2*	365	651	506	143	0.625	1041
*ND3*	145	212	171	41	0.594	357
*ND4*	540	801	644	157	0.584	1371
*ND4L*	133	179	134	44	0.574	312
*ND5*	719	1050	801	246	0.582	1805
*ND6*	159	403	340	59	0.691	583

Most tRNA genes were folded into a cloverleaf secondary structure using MITOS, except for trnS, which lacked the DHU arm in nine species of skipper. Owing to the lack of the DHU stem, trnS was consistently the shortest among the tRNAs of the skippers (Supplementary Fig. S2). The two rRNA genes (rrnL and rrnS) encoding the small and large ribosomal subunits were located between trnL and trnV, as well as trnV and the AT-rich region, respectively.

**Figure 3:**
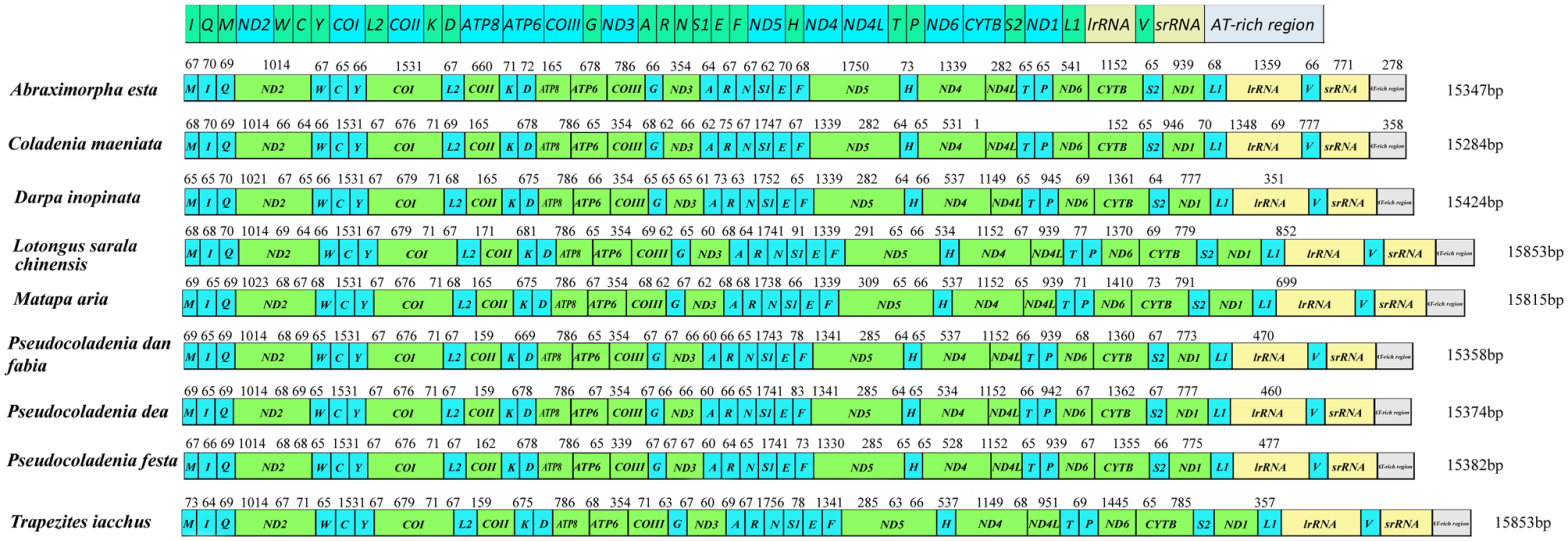
Characters of the nine sequenced hesperiid species’ mitochondrial genomes. Genetic names are replaced by abbreviations.

The AT-rich region, also called the control region, exerts an important function in initiating replication in invertebrates. However, the control region does not encode any known functional genes^26,27^. In our study, the length of the AT-rich region in the nine skipper species ranged from 351 bp (*Darpa inopinata*) to 852 bp (*Lotongus saralus chinensis*). In addition to AT-rich regions, non-coding regions commonly exist between two genes, called spacer regions. Generally, butterflies have a 60–90 bp interval between the mitochondrial genes trnQ and ND2, which may be caused by gene rearrangement^22^. In terms of mitochondrial genome rearrangement, most mitochondrial genomes of Lepidoptera are reported to have trnM rearrangements^17,22,28^; here, the original trnI-trnQ-trnM was rearranged to trnM-trnI-trnQ (Fig. 3).

### Phylogenetic relationships

In this study, 58 species were analyzed based on 37 mitochondrial genes. The total concatenated alignment length after removing ambiguous regions was 15,066 bp.

Four datasets yielded mostly congruent results, differing only in the position of Eudaminae and Pyrginae; we used the results based on the PRT datasets for further analyses. PartitionFinder suggested 53 partitions as optimal for the PRT datasets (Supplementary file S1).

**Figure 4:**
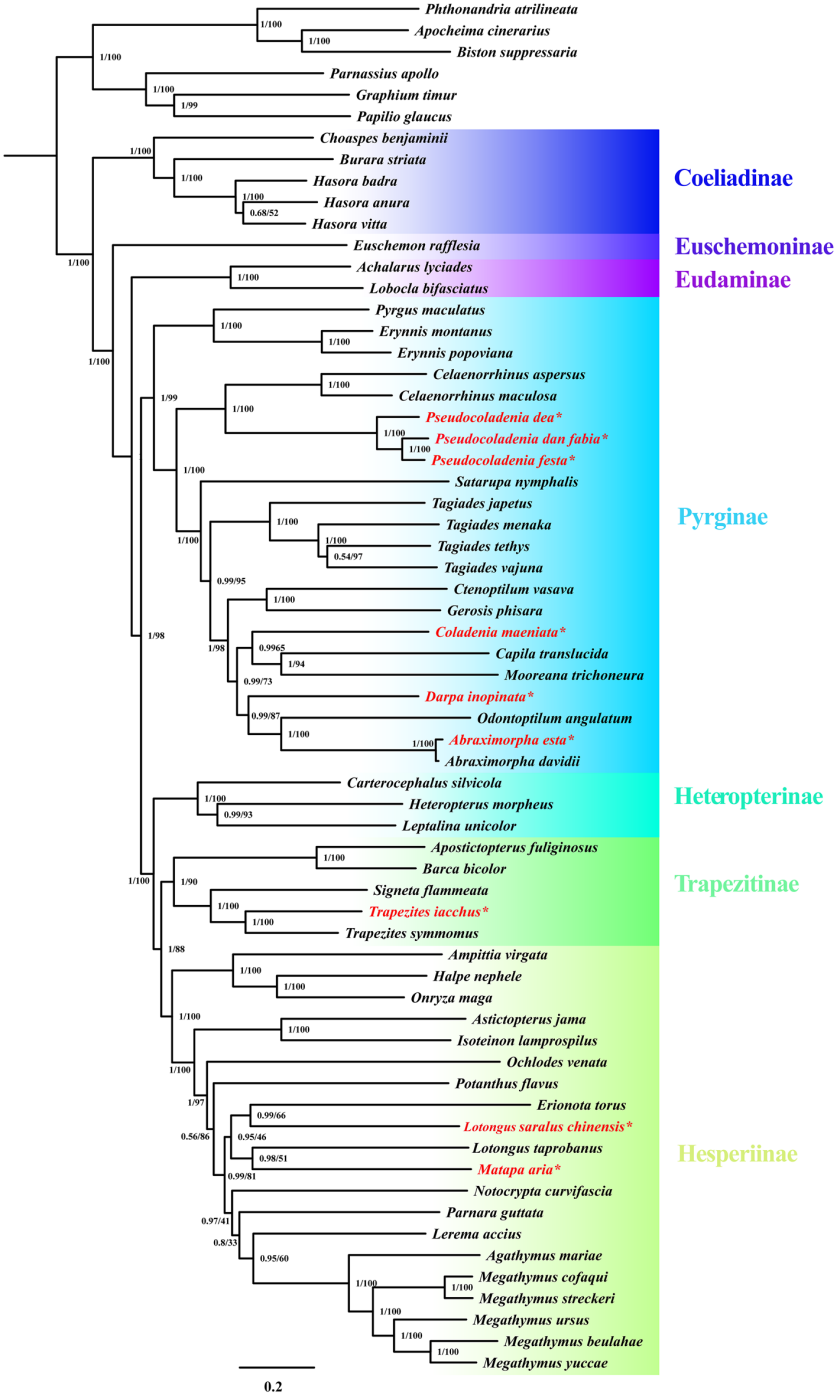
Phylogenetic tree based on PRT dataset. Numbers at node indicates posterior probabilities (PP) and bootstrap value (UFBoot) based on ML analyses were also given. * represents the newly sequenced species.

The phylogenetic trees reconstructed using the ML and BI methods result in an identical topology with strong support (Fig. 4); indicating that Hesperiidae is a strongly supported monophyletic group. Within this family, seven major clades correspond to seven subfamilies: Coeliadinae, Euschemoninae, Eudaminae, Pyrginae, Heteropterinae, Trapezitinae, and Hesperiinae. Similarly to previous studies^4,7,9,16,17,29^, (1) Coeliadinae was branched out at the base of the family; (2) Euschemoninae was sister to the other Hesperiidae excluding Coeliadinae; and (3) Pyrginae sensu lato was a monophyletic group. Within the subfamily Pyrginae, tribes Tagiadini and Celaenorrhinini were sister groups. Li et al. divided Pyrginae sensu lato into three subfamilies: Tagiadinae, Pyrrhopyginae, and Pyrginae^7^. In the current study we did not follow this taxonomy for two reasons. First, we lacked sufficient mitogenomic data for more material. Second, such fragmentation of subfamily opposes the purpose of taxonomically robust and stable classification. Notably, phylogeny of mitogenomes is not equal to the phylogeny of organisms; which are better represented by their nuclear genomes^30,31^. Morphological features, such as venation and genitalia, are encoded in the nuclear genome. Therefore, we also illustrated morphological characters, instead of the phylogenetic tree, and the information of nuclear genome was implicitly used in this study and projected onto the mitochondrial genome phylogeny. Herein, we discuss the following taxonomic problems in subfamily and species level.

**Figure 5:**
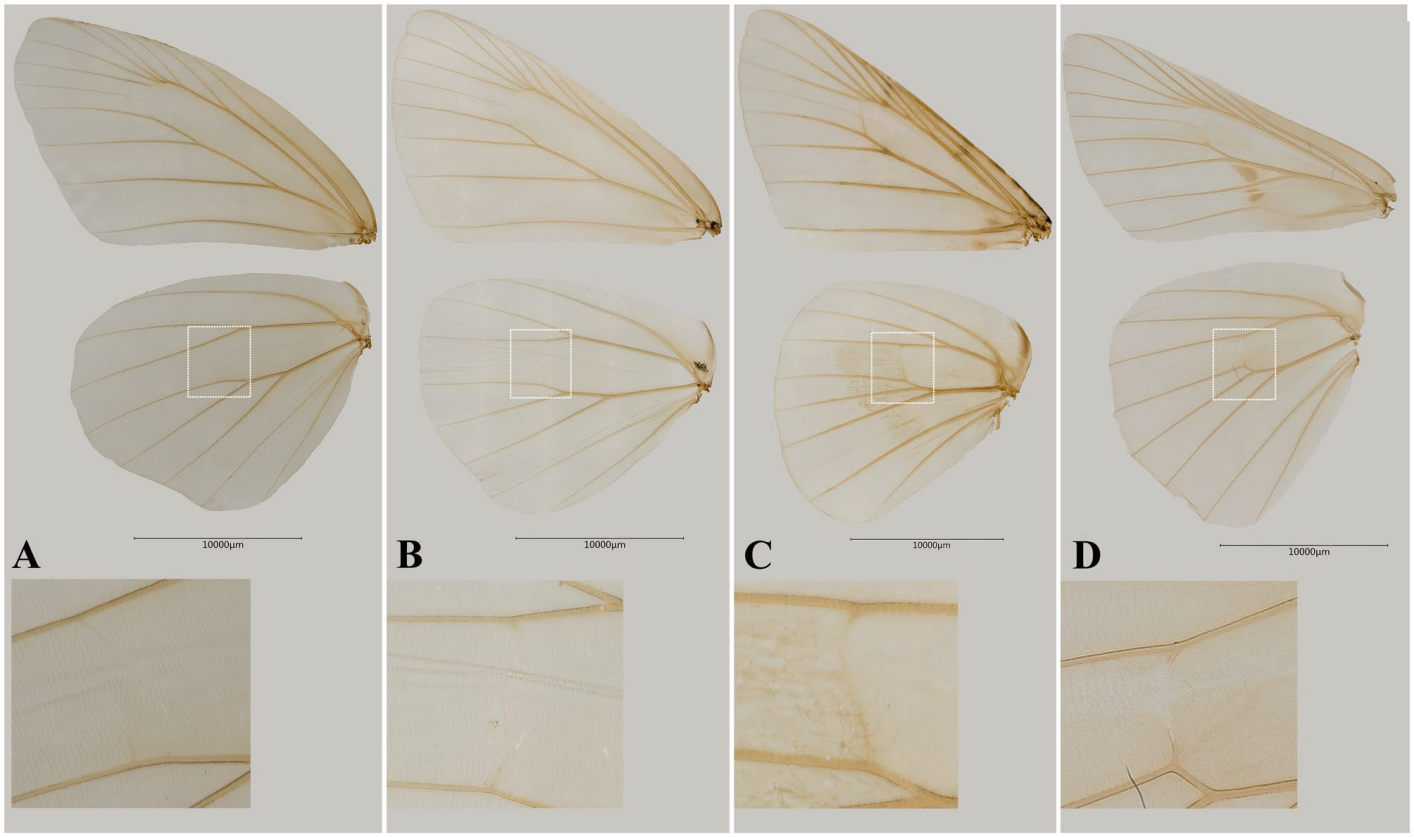
Wing venation of four genera of Trapezitinae. (**A**) *Apostictopterus fuliginosus* (**B**) *Barca bicolor* (**C**) *Trapezites symmomus* (**D**) *Signeta flammeata*.

*Apostictopterus* and *Barca.* Our molecular phylogeny indicates that the Trapezitinae and the clade composed of two Asian genera, *Apostictopterus* and *Barca,* are strongly supported monophyletic groups (PP = 1, UFBoot = 90), which is consistent with the finding of Zhang et al.^8^ These results suggest that the lineage of *Apostictopterus* and *Barca* belong to the subfamily Trapezitinae or represent a separate subfamily. Zhang et al.^8^ claimed that no apparent morphological synapomorphies unified the group of the two genera with the Trapezitinae and treated the lineage as a separate subfamily, Barcinae. Nevertheless, we did not follow this classification. Instead, we classified them as a tribe, Barcini stat. nov., within the subfamily Trapezitinae. The Trapezitinae in conventional sense is a monophyletic group which has been characterized by single synapomorphy: the discocellular vein on the hindwing is directed toward the wing apex^14^. Braby noted that the apex of the hindwing cell, in the Trapezitinae, is truncated, with the discocellular vein between M_2_ and M_3_ angled or inclined toward the dorsum, and that vein M_2_ is always present but usually weakly developed in the Trapezitinae^32^. Our morphological study also demonstrates that vein 1A + 2A on the forewing is a bow-like in shape that changes gradually: faintly curved in *Trapezites* (Fig. 5C), moderately curved in *Atkinsia*^33^, and prominently arched in *Signeta* (Fig. 5D). Similarly, Zhang et al.^8^ proposed the subfamily Barcinae, characterized by a vein 1A + 2A bow-like shape on the forewing. *Apostictopterus* and *Barca* share similar characteristics of the discocellular vein and weakly developed vein M_2_ on the hindwing and bow-like shaped vein 1A + 2A on the forewing with genera of the Trapezitinae (Fig. 5). During immature stages, eggs of *Apostictopterus* and *Barca*, like most eggs of the Trapezitinae, are strongly ribbed^33^. Biogeographically distributions such as this one in southern China and Australia are also found in Miletinae. Both *Apostictopterus* and *Barca* are distributed throughout Asia, while all the Trapezitinae members are distributed in Australia and adjacent locations. These disjunct distributions (e.g., southern China and Australia) were also identified in Miletinae, Lycaenidae^34,35^. Alternative taxonomic treatment includes Herteropterinae + Trapezitinae (including *Apostictopterus* and *Barca*) + Hesperiinae as a single subfamily Hesperiinae (PP = 1, UFBoot = 100) or Trapezitinae (including *Apostictopterus* and *Barca*) + Hesperiinae, excluding Heteropterinae, as a subfamily Hesperiinae (PP = 1, UFBoot = 88). Further extensive examination of morphology, life history and other biological data will increase our ability to determine which taxonomic treatment is the most suitable taxonomic framework.

*Pseudocoladenia dea* and *P. festa*. In our study, three members of the genus *Pseudocoladenia*, *P. dea* (from Yingjing*,* Sichuan), *P. festa* (from Moxi, Sichuan), and *P. dan fabia* (from Yingde, Guangdong), were clustered into a highly supported clade, and *P. dea* was a sister to *P. festa* + *P. dan fabia*. Male genitalia differ among these taxa. In *P. dea*, the valva had a ventrodistal process vertically elongated (long and pointed distally in *P. festa* and short and small in *P. dan fabia*) and the aedeagus possesses a dorsal spiny process (no such process in the other two).

The genetic distance was calculated for the COI barcoding region (658 bp), and the results indicated that the distance between *P. dea* and *P. dan fabia* was 4.9%, 3.7% between *P. festa* and *P. dan fabia*, and 4.7% between *P. dea* and *P. festa*. Although we could not sample the nominate subspecies *P. dan dan* (type locality: Tranquebar, India), *P. dan fabia* and the nominate subspecies in our study shared similar wing patterns and male genitalia. Thus, it appears appropriate to discuss the status of *P. dea* and *P. festa* with *P. dan fabia.*

Recently, Huang restored the subspecies of *P. dan*, *fatih* (type locality: Mussoorie, India), to species level and classified *P. festa* as a subspecies of *P. fatih* because of its allopatric distribution to *P. festa* and a lack of differences in their male genitalia^36^. Further, based on the distributions of *P. fatih* and *P. festa* in India (https://www.ifoundbutterflies.org/pseudocoladenia-fatih or festa, Anonymous 2022) as well as our specimens from Zhang Mu, Tibet, the two taxa appear sympatric. In addition, *P. fatih* can be morphologically distinguished from *P. festa* based on differences in the white sub-hyaline spots on the forewing and the arc-shaped outward edge of the ventrodistal process of valva (Huang, 2021, Fig. 85: 22-1, 23-1). Lastly, we obtained a 658 bp COI sequence for *P. fatih* and estimated the genetic distance between *P. fatih* and *P. festa* to be 2.3%. Based on the differences in wing pattern and male genitalia, with supporting molecular data, we suggest that all *P. dea*, *P. fatih,* and *P. festa* should be regarded as distinct species.

**Figure 6:**
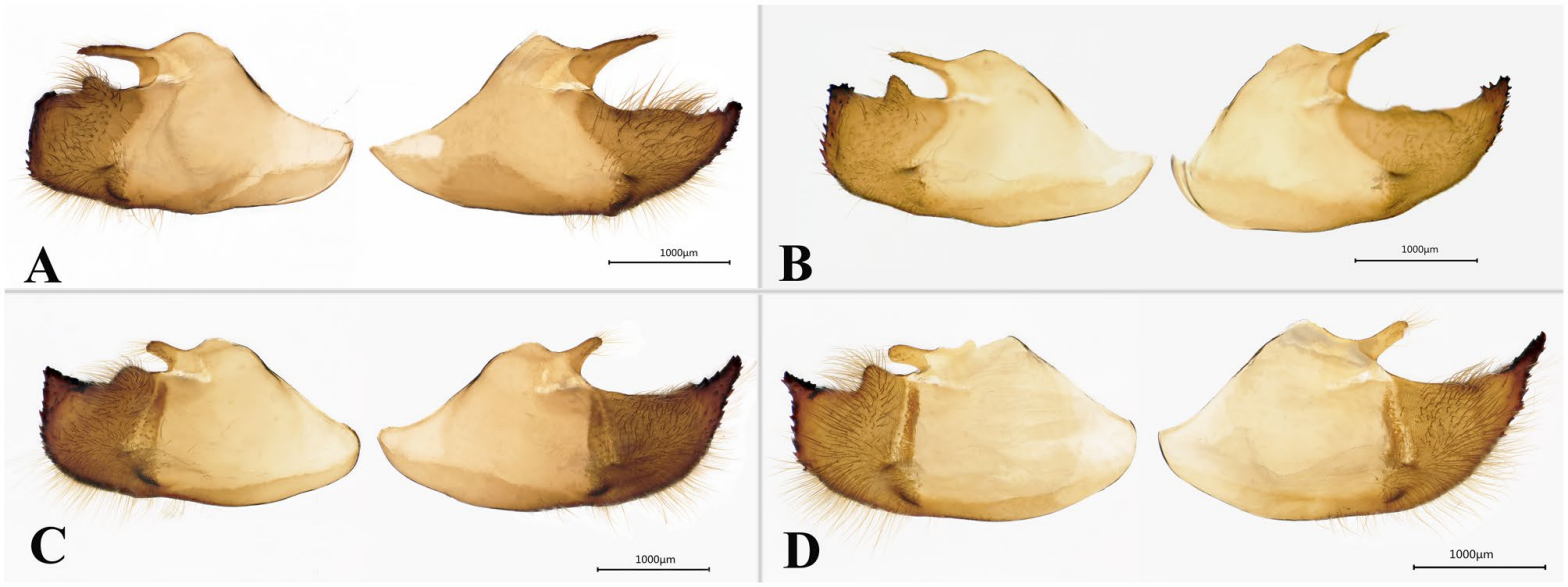
Valvae of two *Abraximorpha* skippers. (**A**,**B**) *A.esta* (A: Baoshan, Yunnan; B: Vietnam);. (**C**, **D**) *A.davidii* (C:Baoxin, Sichuan; D: Chongqing).

*Abraximorpha esta.* Evans described *A. esta* as a subspecies of *A. davidii*^12^*.* Devyatkin & Monastyrskii^37^, however, regarded *A. esta* as a separate species based on morphological examination of the type specimens stored in the Natural History Museum, London. In this study, *A. esta* and *A. davidii* were recovered as sister taxa. The COI-based sequence divergence between these two taxa was 0.9%. Genetic divergence values between closed sister taxa are often lower than 1%, which is very common in Lepidoptera because hybridization, introgression or incomplete lineage sorting can cause low interspecific divergence or produce young species in which divergence is too recent for lineage sorting to complete^38–40^. Our morphological study showed that *A. esta* can be distinguished from *A. davidii* based on the following characteristics: In *A. esta*, the wing pattern on the dorsal side is the same as that on the ventral side, the spots at the apex are radial, and the spots in spaces CuA1 and CuA2 are long-rectangular shaped. In contrast, in *A. davidii*, white spots on the ventral side are more developed than those of the dorsal side, the spots at the apex are small and rectangular, and only the spot in the space CuA_1_ is long-rectangular shaped. The male genitalia show significant differences between these two taxa (Fig. 6): in *A. esta*, the dorsal process in the middle of valva is long, thin, and pointed, reaching dorsal swelling and its ventrodistal process with the inner edge is S-shaped, whereas in *A. davidii*, the dorsodistal process is short with a blunt tip, not reaching the swelling in left valva; the ventrodistal process of the inner edge is straight, although there are individual variations in *A. davidii*. In summary, based on the difference in wing pattern and male genitalia described above combined with the work of Devyatkin and Monastyrskii^37^ and Osada et al.^41^ suggesting their sympatric distribution, we agree with Devyatkin & Monastyrskii^37^ that *A. esta* is a distinct species.

**Figure 7:**
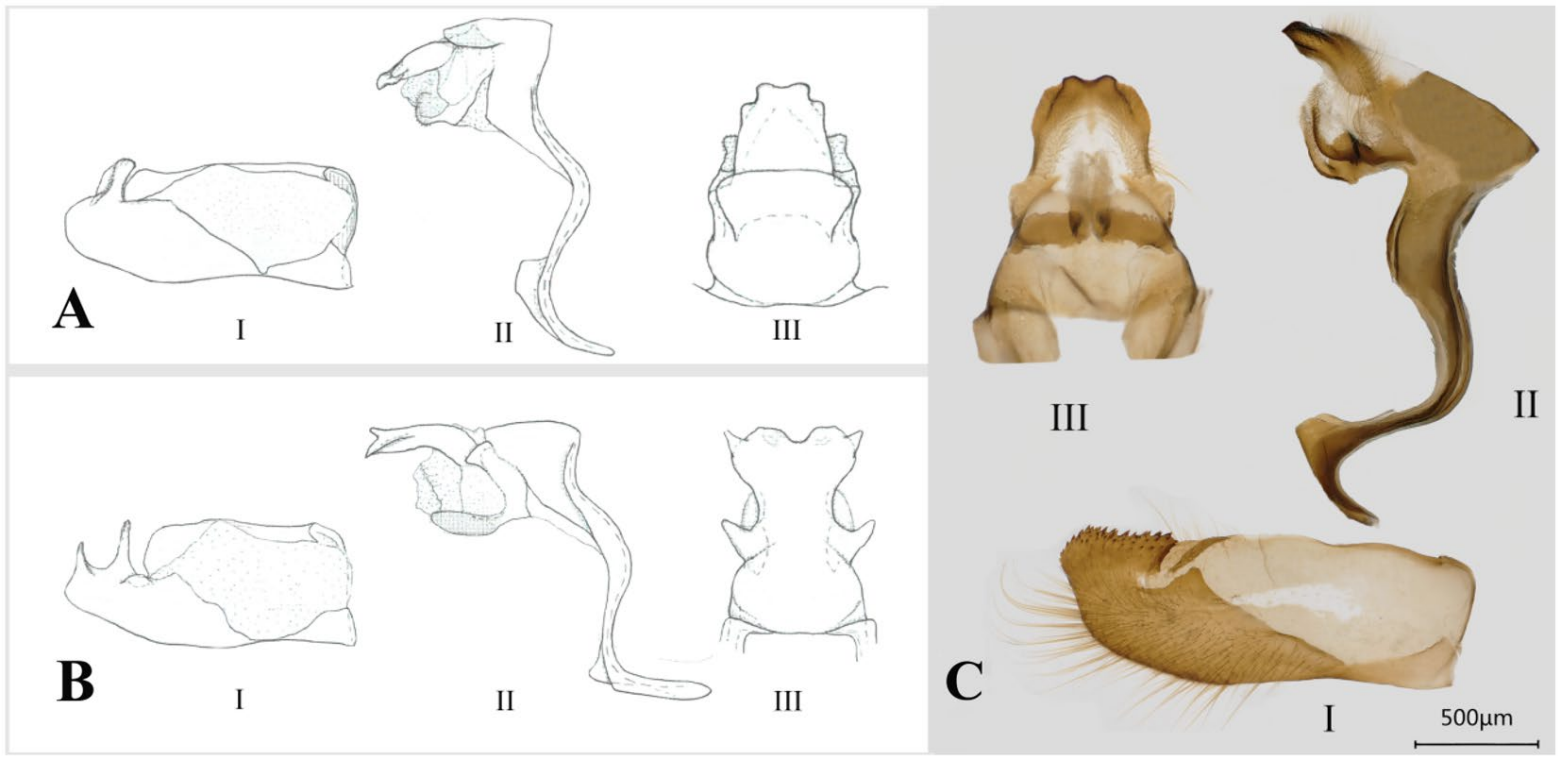
Male genitalia of *Acerbas* and *Lotongus*. (**A**) *A. anthea*; (**B**) *L.calathus calathus*; (**C**) *L. saralus chinensis*. I: Left valva, inner view; II: Genitalia ring, lateral view; III: Tegument, dorsal view.

*Acerbas saralus chinensis* comb. nov.. Cong et al.^2^ and Zhang et al.^9^ reclassified *Lotongus saralus* into the genus *Acerbas* based on genomic phylogenetic analyses of the nominate subspecies. Our phylogenetic analyses also indicated that the genus *Lotongus* is not monophyletic. Further, in our phylogeny, *Lotongus saralus chinensis* was a sister to *Erionota*, whereas *Lotongus taprobanus*^42^ was closely related to *Matapa*. The male genitalia of *L. calathus* differed from those of *L. saralus,* including a pair of lateral cone-shaped process of dorsum and apically broad uncus, with small and pointed dorso-lateral processes (Fig. 7), implying that *Lotongus* is not a monophyletic group. Although we failed to sample the type species of *Acerbas* and the nominate subspecies of *L. saralus* in this study, *L. saralus chinensis* exhibited a similarity in male genitalia implying its close relationship with *Acerbas*: the socius is absent and the uncus is shallowly indented apically (Fig. 7A,C), resembling its nominate subspecies in terms of wing pattern and male genitalia. Therefore, based on our morphological and molecular evidence, as well as the results of Cong et al.^2^ and Zhang et al.^9^, we agree that *Lotongus saralus* was misclassified in *Lotongus* and should be reclassified to the genus *Acerbas* along with the subspecies *chinensis*.

## Conclusion

Stable phylogenetic relationships are key to understanding the role of speciation in the formation of biodiversity. In this study, a phylogenetic tree was constructed based on mitogenome data and morphological analysis. We suggest that (1) *Barca* and *Apostictopterus* should be reclassified together as a tribe Barcini in the subfamily Trapezitinae; (2) *Pseudocoladenia festa*, *P. dea*, and *Abraximorpha esta* are distinct species and; (3) *Lotongus saralus chinensis* should be transferred to the genus *Acerbas*. Although this study shares similarities with previously published research^3,8,17,24^, our results were based on a molecular phylogenetic analysis, integrated morphological characters and Bayesian statistics which allowed us to better evaluate the state of these groups. Thus, this study confirms the results of previous studies, thereby improving confidence in the respective suggestions for taxonomic rank. Additionally, mtDNA data alone cannot identify issues on different taxonomical levels. In the study of the Hesperiidae, information from morphology is vital for classification at different levels. Finally, in some branches of the phylogenetic tree, there were no high node support values. These issues will be addressed in future studies, adding nuclear genomic data and additional materials to reveal the phylogenetic relationships within the family Hesperiidae.

## Materials and methods

### Taxon sampling and morphological study

Thirteen species belonging to eight genera of the family Hesperiidae were sampled (Supplementary Table S3). Among these, whole-genome data of four species were available at NCBI (*Tagiades japentus*,* T*. *symmonus*, *Signeta flammeata*, and *Lotongus calathus taprobanus*). Seven species were sequenced for the first time, including *Pseudocoladena festa*, *P. dea*, *Darpa inopinata*, *Abraximorpha esta*, *Coladenia maeniata*, *Trapezites iacchus*, and *L. saralus chinensis*. Additionally, *P. dan fabia* and *Matapa aria* have been sequenced in previous studies^9,29^; however, the collection locality differed from that in this study. The specimens used in this study were stored at South China Agricultural University (SCAU), Guangzhou, China. Methods of morphological studies of adult specimens and male genitalia described Fan et al. were followed^43^, and the wing venation treatment was performed according to the method outlined by Hou et al.^44^.

### Laboratory protocols

Total DNA was extracted from the leg muscle tissue of a single adult butterfly for each sample using the HiPure Insect DNA Kit (Magen, China), following the manufacturer’s instructions. For amplification, complete mitogenomes were divided into 27 overlapping fragments. The primers and amplification fragment steps were performed as described by Kim et al.^25^ and Han et al.^17^. Owing to the AT-rich region instability, we cloned this fragment, using methods outlined by Fan et al.^45^, after amplification.

### Mitogenome annotation and data analyses

The sequences obtained were proofread and then assembled using the software Geneious v7.1.4^46^. Protein-coding genes (PCGs) were identified by finding the respective ORFs on the NCBI website (https://www.ncbi.nlm.nih.gov/oreeinder) with the invertebrate mitochondrial genetic codes. Next, tRNAs and rRNAs were identified using the MITOS Web Server (http://mitos.bioinf.uni-leipzig.de/index.py). Each new sequence was aligned against the complete mitogenomes of other skippers using MAFFT v7.313^47^ to determine gene boundaries and relative positions. The AT-rich region was identified by recognizing the boundaries between *rrn*S and *trn*M. Nucleotide composition bias was calculated using the formula: AT-skew = (A − T)/(A + T); GC-skew = (G − C)/(G + C)^48^.

### Phylogenetic analysis

The ingroup for phylogenetic analysis included 13 sequences from this study and 45 publicly available mitogenomes, collectively representing 58 hesperiid species (Supplementary Table S1). The mitogenomes of six species were obtained from GenBank as outgroup (Supplementary Table S1).

Each of the 13 PCGs was aligned individually using the software MAFFT V7.3.13^49^. Specifically, tRNAs and rRNAs were aligned separately using the Q-INS-i strategy through the MAFFT V7.313 online alignment server (https://maf.cbrc.jp/alignment/server/)^50^. Gaps and ambiguous sites from the 13 PCGs were removed using the Gblocks V0.91 online server (http://molevol.cmima.csic.es/castresana/Gblocks_server.html).

We reconstructed ML and BI tree of four datasets (PRT, PCGD, PCGC, and PCGR)^14^, in which we used the partition scheme produced by PartitionFinder v2.1.1 under the Bayesian information criterion (BIC)^47^. ML analyses were performed on the IQ-TREE web online server (http://iqtree.cibiv.univie.ac.at/, accessed in May 2022)^51^ with 1000 ultrafast bootstraps (UFBoot) to estimate branch support. We set bootstrap analysis to ultrafast and the number of bootstrap alignments to 1000, UFBoot indicates the confidence level of each branch, generally considered to be high when UFBoot ≥ 95^52^, and the rest were use as default parameters. BI analyses were performed using MrBayes V3.2.6 on the CIPRES Science Gateway 3.3 (http://www.phylo.org/)^53^. The phylogenetic tree construction model setting adopts reversible jump Markov chain Monte Carl (MCMC), the specific parameter settings: set 4 MCMC chains (1 cold chain and 3 hot chains) each chain ran 5 × 10^8^ generations, sampling once every 1 × 10^3^ generations, running independently twice, discarding the 25% aging tree that just runs. The file obtained after running uses Tracer V1.6^54^ to verify whether the ESS value of each valid sample size was greater than 200, that is, whether Bayesian converges. The phylogenetic tree is shown with FigTree v1.4.3. Overall, this study is almost identical to methods used by Han et al.^17^, with the only difference being our larger sample size with additional species. In this current study, the genetic distances for the COI barcodes were calculated using Kamar 2–parameter model in MEGA X^55^.

## Supplementary Information

Supplementary Information 1.

Supplementary Information 2.

## Data Availability

The data that support the findings of this study are available from [National Center for Biotechnology Information]. We have provided GenBank accession numbers: OP723917-OP723926, OQ784637-OQ784639.
