# Supplementary material for: Mitogenomic phylogenetic analyses provide novel insights into the taxonomic problems of several hesperiid taxa (Lepidoptera: Hesperiidae)
Source: Sci Rep. 2023 May 16;13:7901. doi: 10.1038/s41598-023-34608-8 (PMC10188531; doi:10.1038/s41598-023-34608-8)
Supplement: Supplementary file 1 — Supplementary Information 1. [file 41598_2023_34608_MOESM1_ESM.docx]

**Supplementary file 1.** PRT data partitioning (a) and PartitionFinder compute the optimal model (b.c).

| **a. Data partitioning** | | |
| --- | --- | --- |
| ND2= 1-1024; | trnM= 10937-11019; | trnN= 11823-11894; |
| CO1= 1025-2558; | trn1= 11020-11091; | trnS1= 11895-11961; |
| CO11 = 2559-3240; | trnQ= 11092-11164; | trnE= 11962-12037; |
| ATP8 = 3241-3444; | trnW = 11165-11237; | trnF= 12038-12109; |
| ATP6= 3445-4143; | trnC= 11238-11313; | trnH= 12110-12186; |
| CO111= 4144-4936; | trnY= 11314-11387; | trnT= 12187-12254; |
| ND3= 4937-5290; | trnL2= 11388-11456; | trnP= 12255-12322; |
| ND5= 5291-7051; | trnK= 11457-11527; | trnS2= 12323-12395; |
| ND4= 7052-7976; | trnD= 11528-11604; | trnL1 = 12396-12473; |
| ND4L = 7977-8267; | trnG= 11605-11676; | trnV = 12474-12549; |
| ND6= 8268-8835; | trnA= 11677-11749; | lrRNA= 12550-14178; |
| CYTB = 8836-9990; | trnR= 11750-11822; | srRNA= 14179-15066; |
| ND1= 9991-10936; |  |  |

**b. ML tree optimal model**

#nexus

begin sets;

charset Subset1 = 1-1024\3;

charset Subset2 = 2-1024\3;

charset Subset3 = 3-1024\3;

charset Subset4 = 1025-2558\3;

charset Subset5 = 1026-2558\3;

charset Subset6 = 4144-4936\3 1027-2558\3;

charset Subset7 = 11458-11527\3 2559-3240\3;

charset Subset8 = 8837-9990\3 2560-3240\3;

charset Subset9 = 2561-3240\3;

charset Subset10 = 3241-3444\3;

charset Subset11 = 3242-3444\3 12040-12109\3;

charset Subset12 = 5292-7051\3 3243-3444\3;

charset Subset13 = 3445-4143\3;

charset Subset14 = 3446-4143\3;

charset Subset15 = 3447-4143\3 9991-10936\3;

charset Subset16 = 4145-4936\3 11459-11527\3;

charset Subset17 = 4146-4936\3;

charset Subset18 = 11092-11164\3 11390-11456\3 4937-5290\3 11166-11237\3 11093-11164\3;

charset Subset19 = 4938-5290\3;

charset Subset20 = 4939-5290\3 8838-9990\3;

charset Subset21 = 12476-12549\3 5291-7051\3;

charset Subset22 = 5293-7051\3;

charset Subset23 = 7052-7976\3 11314-11387\3;

charset Subset24 = 7053-7976\3;

charset Subset25 = 7054-7976\3 14179-15066\3;

charset Subset26 = 9992-10936\3 7977-8267\3;

charset Subset27 = 7978-8267\3 10939-11019\3;

charset Subset28 = 11240-11313\3 7979-8267\3;

charset Subset29 = 12039-12109\3 11962-12037\3 8268-8835\3;

charset Subset30 = 8269-8835\3;

charset Subset31 = 8270-8835\3;

charset Subset32 = 8836-9990\3;

charset Subset33 = 9993-10936\3;

charset Subset34 = 10937-11019\3 11528-11604\3 11752-11822\3;

charset Subset35 = 11388-11456\3 10938-11019\3;

charset Subset36 = 11751-11822\3 11020-11091\3 11895-11961\3 12187-12254\3;

charset Subset37 = 11021-11091\3 12255-12322\3 11963-12037\3 12188-12254\3 11607-11676\3;

charset Subset38 = 11165-11237\3 11022-11091\3;

charset Subset39 = 11094-11164\3 11389-11456\3;

charset Subset40 = 11239-11313\3 11457-11527\3 11167-11237\3;

charset Subset41 = 11896-11961\3 12110-12186\3 11238-11313\3;

charset Subset42 = 12474-12549\3 11315-11387\3;

charset Subset43 = 11606-11676\3 11964-12037\3 11316-11387\3 11530-11604\3;

charset Subset44 = 11678-11749\3 11825-11894\3 11750-11822\3 11529-11604\3 11823-11894\3;

charset Subset45 = 12323-12395\3 12324-12395\3 11605-11676\3 11679-11749\3;

charset Subset46 = 12111-12186\3 11677-11749\3 12112-12186\3;

charset Subset47 = 11824-11894\3 12256-12322\3 11897-11961\3 12397-12473\3;

charset Subset48 = 12038-12109\3;

charset Subset49 = 12189-12254\3 12398-12473\3;

charset Subset50 = 12257-12322\3 12325-12395\3 12475-12549\3;

charset Subset51 = 12396-12473\3;

charset Subset52 = 12551-14178\3 12552-14178\3 12550-14178\3;

charset Subset53 = 14181-15066\3 14180-15066\3;

charpartition PartitionFinder = GTR+I+G:Subset1, GTR+I+G:Subset2, GTR+I+G:Subset3, GTR+I+G:Subset4, GTR+I+G:Subset5, GTR+I+G:Subset6, GTR+I+G:Subset7, GTR+I+G:Subset8, GTR+I+G:Subset9, GTR+I+G:Subset10, GTR+I+G:Subset11, GTR+I+G:Subset12, GTR+I+G:Subset13, GTR+I+G:Subset14, GTR+I+G:Subset15, GTR+I+G:Subset16, GTR+I+G:Subset17, GTR+I+G:Subset18, GTR+I+G:Subset19, GTR+I+G:Subset20, GTR+I+G:Subset21, GTR+I+G:Subset22, GTR+I+G:Subset23, GTR+I+G:Subset24, GTR+I+G:Subset25, GTR+I+G:Subset26, GTR+I+G:Subset27, GTR+I+G:Subset28, GTR+I+G:Subset29, GTR+I+G:Subset30, GTR+G:Subset31, GTR+I+G:Subset32, GTR+I+G:Subset33, GTR+I+G:Subset34, GTR+G:Subset35, GTR+G:Subset36, GTR+I+G:Subset37, GTR+G:Subset38, GTR+G:Subset39, GTR+G:Subset40, GTR+G:Subset41, GTR+G:Subset42, GTR+I+G:Subset43, GTR+I+G:Subset44, GTR+G:Subset45, GTR+G:Subset46, GTR+I+G:Subset47, GTR+G:Subset48, GTR+G:Subset49, GTR+G:Subset50, GTR+G:Subset51, GTR+I+G:Subset52, GTR+I+G:Subset53;

end;

**c. BI tree optimal model**

begin mrbayes;

charset Subset1 = 1-1024\3;

charset Subset2 = 2-1024\3;

charset Subset3 = 3-1024\3;

charset Subset4 = 1025-2558\3;

charset Subset5 = 1026-2558\3;

charset Subset6 = 4144-4936\3 1027-2558\3;

charset Subset7 = 11458-11527\3 2559-3240\3;

charset Subset8 = 8837-9990\3 2560-3240\3;

charset Subset9 = 2561-3240\3;

charset Subset10 = 3241-3444\3;

charset Subset11 = 3242-3444\3 12040-12109\3;

charset Subset12 = 5292-7051\3 3243-3444\3;

charset Subset13 = 3445-4143\3;

charset Subset14 = 3446-4143\3;

charset Subset15 = 3447-4143\3 9991-10936\3;

charset Subset16 = 4145-4936\3 11459-11527\3;

charset Subset17 = 4146-4936\3;

charset Subset18 = 11092-11164\3 11390-11456\3 4937-5290\3 11166-11237\3 11093-11164\3;

charset Subset19 = 4938-5290\3;

charset Subset20 = 4939-5290\3 8838-9990\3;

charset Subset21 = 12476-12549\3 5291-7051\3;

charset Subset22 = 5293-7051\3;

charset Subset23 = 7052-7976\3 11314-11387\3;

charset Subset24 = 7053-7976\3;

charset Subset25 = 7054-7976\3 14179-15066\3;

charset Subset26 = 9992-10936\3 7977-8267\3;

charset Subset27 = 7978-8267\3 10939-11019\3;

charset Subset28 = 11240-11313\3 7979-8267\3;

charset Subset29 = 12039-12109\3 11962-12037\3 8268-8835\3;

charset Subset30 = 8269-8835\3;

charset Subset31 = 8270-8835\3;

charset Subset32 = 8836-9990\3;

charset Subset33 = 9993-10936\3;

charset Subset34 = 10937-11019\3 11528-11604\3 11752-11822\3;

charset Subset35 = 11388-11456\3 10938-11019\3;

charset Subset36 = 11751-11822\3 11020-11091\3 11895-11961\3 12187-12254\3;

charset Subset37 = 11021-11091\3 12255-12322\3 11963-12037\3 12188-12254\3 11607-11676\3;

charset Subset38 = 11165-11237\3 11022-11091\3;

charset Subset39 = 11094-11164\3 11389-11456\3;

charset Subset40 = 11239-11313\3 11457-11527\3 11167-11237\3;

charset Subset41 = 11896-11961\3 12110-12186\3 11238-11313\3;

charset Subset42 = 12474-12549\3 11315-11387\3;

charset Subset43 = 11606-11676\3 11964-12037\3 11316-11387\3 11530-11604\3;

charset Subset44 = 11678-11749\3 11825-11894\3 11750-11822\3 11529-11604\3 11823-11894\3;

charset Subset45 = 12323-12395\3 12324-12395\3 11605-11676\3 11679-11749\3;

charset Subset46 = 12111-12186\3 11677-11749\3 12112-12186\3;

charset Subset47 = 11824-11894\3 12256-12322\3 11897-11961\3 12397-12473\3;

charset Subset48 = 12038-12109\3;

charset Subset49 = 12189-12254\3 12398-12473\3;

charset Subset50 = 12257-12322\3 12325-12395\3 12475-12549\3;

charset Subset51 = 12396-12473\3;

charset Subset52 = 12551-14178\3 12552-14178\3 12550-14178\3;

charset Subset53 = 14181-15066\3 14180-15066\3;

partition PartitionFinder = 53:Subset1, Subset2, Subset3, Subset4, Subset5, Subset6, Subset7, Subset8, Subset9, Subset10, Subset11, Subset12, Subset13, Subset14, Subset15, Subset16, Subset17, Subset18, Subset19, Subset20, Subset21, Subset22, Subset23, Subset24, Subset25, Subset26, Subset27, Subset28, Subset29, Subset30, Subset31, Subset32, Subset33, Subset34, Subset35, Subset36, Subset37, Subset38, Subset39, Subset40, Subset41, Subset42, Subset43, Subset44, Subset45, Subset46, Subset47, Subset48, Subset49, Subset50, Subset51, Subset52, Subset53;

set partition=PartitionFinder;

lset applyto=(1) nst=6 rates=invgamma;

lset applyto=(2) nst=6 rates=invgamma;

lset applyto=(3) nst=6 rates=invgamma;

lset applyto=(4) nst=6 rates=invgamma;

lset applyto=(5) nst=6 rates=invgamma;

lset applyto=(6) nst=6 rates=invgamma;

lset applyto=(7) nst=6 rates=invgamma;

lset applyto=(8) nst=6 rates=invgamma;

lset applyto=(9) nst=6 rates=invgamma;

lset applyto=(10) nst=6 rates=invgamma;

lset applyto=(11) nst=6 rates=invgamma;

lset applyto=(12) nst=6 rates=invgamma;

lset applyto=(13) nst=6 rates=invgamma;

lset applyto=(14) nst=6 rates=invgamma;

lset applyto=(15) nst=6 rates=invgamma;

lset applyto=(16) nst=6 rates=invgamma;

lset applyto=(17) nst=6 rates=invgamma;

lset applyto=(18) nst=6 rates=invgamma;

lset applyto=(19) nst=6 rates=invgamma;

lset applyto=(20) nst=6 rates=invgamma;

lset applyto=(21) nst=6 rates=invgamma;

lset applyto=(22) nst=6 rates=invgamma;

lset applyto=(23) nst=6 rates=invgamma;

lset applyto=(24) nst=6 rates=invgamma;

lset applyto=(25) nst=6 rates=invgamma;

lset applyto=(26) nst=6 rates=invgamma;

lset applyto=(27) nst=6 rates=invgamma;

lset applyto=(28) nst=6 rates=invgamma;

lset applyto=(29) nst=6 rates=invgamma;

lset applyto=(30) nst=6 rates=invgamma;

lset applyto=(31) nst=6 rates=gamma;

lset applyto=(32) nst=6 rates=invgamma;

lset applyto=(33) nst=6 rates=invgamma;

lset applyto=(34) nst=6 rates=invgamma;

lset applyto=(35) nst=6 rates=gamma;

lset applyto=(36) nst=6 rates=gamma;

lset applyto=(37) nst=6 rates=invgamma;

lset applyto=(38) nst=6 rates=gamma;

lset applyto=(39) nst=6 rates=gamma;

lset applyto=(40) nst=6 rates=gamma;

lset applyto=(41) nst=6 rates=gamma;

lset applyto=(42) nst=6 rates=gamma;

lset applyto=(43) nst=6 rates=invgamma;

lset applyto=(44) nst=6 rates=invgamma;

lset applyto=(45) nst=6 rates=gamma;

lset applyto=(46) nst=6 rates=gamma;

lset applyto=(47) nst=6 rates=invgamma;

lset applyto=(48) nst=6 rates=gamma;

lset applyto=(49) nst=6 rates=gamma;

lset applyto=(50) nst=6 rates=gamma;

lset applyto=(51) nst=6 rates=gamma;

lset applyto=(52) nst=6 rates=invgamma;

lset applyto=(53) nst=6 rates=invgamma;

mcmcp ngen= 10000000 relburnin=yes burninfrac=0.25 printfreq=1000 samplefreq=1000 nchains=4 savebrlens=yes;

mcmc;

sumt;

End;
