# Supplementary material for: Mitogenomic phylogenetic analyses provide novel insights into the taxonomic problems of several hesperiid taxa (Lepidoptera: Hesperiidae)
Source: Sci Rep. 2023 May 16;13:7901. doi: 10.1038/s41598-023-34608-8 (PMC10188531; doi:10.1038/s41598-023-34608-8)
Supplement: Supplementary file 2 — Supplementary Information 2. [file 41598_2023_34608_MOESM2_ESM.docx]

**Mitogenomic phylogenetic analyses provide novel insights into the taxonomic problems of several hesperiid taxa (Lepidoptera: Hesperiidae)**

Lijuan Zhu^a,b#^, Yuke Han^c#^, Yongxiang Hou^a#^, Zhenfu Huang^d^, Min Wang^a^, Hideyuki Chiba^e,^*, Liusheng Chen^b,^*& Xiaoling Fan^a^

^a^ Department of Entomology, College of Agriculture, South China Agricultural University, Guangzhou, 510642, China.

^b^ Guangdong Academy of Forestry, Guangzhou 510520, China

^c^ Guangdong Southern Newspaper Media Group Co., Ltd, Guangzhou, 510601, China.

^d^ School of Life Science and Engineering, Southwest University of Science and Technology, Mianyang, 621010, China.

^e^ B. P. Bishop Museum, Honolulu, Hawaii, 96817-0916, USA.

^#^ these three authors contributed equally to this work.

* Correspondence author: Hideyuki Chiba (Email: [chiba.zootaxa@gmail.com](mailto:chiba.zootaxa@gmail.com)) Liusheng Chen (Email: [lshchen2008@163.com)](mailto:lshchen2008@163.com;).

**Supplementary catalogue**

**[Supplementary Table S1.](#_Toc9424)** [The mitochondrial genome sequences of the 58 Hesperiidae and 6 outgroup species used in this study. 1](#_Toc9424)

**[Supplementary Table S2.](#_Toc3451)** [The organization of mitochondrial genomes of nine species. In the column intergenic length, the positive number indicates interval base pairs between genes, while the negative number indicates the overlapping base pairs between genes. 4](#_Toc3451)

**[Supplementary Table S3.](#_Toc14915)** [The collecting information of the specimens used in this study. * denotes the four that have been reported on NCBI, but not directly available to us. So we sequenced the mitochondrial data. 14](#_Toc14915)

**[Supplementary Fig. S1.](#_Toc19459)** [Mitochondrial genome circle map of 9 species. 15](#_Toc19459)

**[Supplementary Fig. S2.](#_Toc10540)** [Predicted secondary clover-leaf structure for the tRNA genes of nine Hesperiidae species sequenced in this study. 24](#_Toc10540)

**[Supplementary file S1](#_Toc2656)**[. PRT data partitioning (a) and PartitionFinder compute the optimal model (b.c). 25](#_Toc2656)

[a. Data partitioning 25](#_Toc11498)

[b. ML tree optimal model 25](#_Toc8780)

[c. BI tree optimal model 27](#_Toc23763)

**Supplementary Table S1.** The mitochondrial genome sequences of the 58 Hesperiidae and 6 outgroup species used in this study.

| Species | Family | Size | accession numbers | Reference |
| --- | --- | --- | --- | --- |
| *Abraximorpha esta* | Hesperiidae | 15, 347 bp | OP723917 | This study |
| *Acerbas sarala chinensis* | Hesperiidae | 15, 853 bp | OP723918 | This study |
| *Achalarus lyciades* | Hesperiidae | 15, 612 bp | NC_030602 | Shen et al., (2016) |
| *Agathymus mariae* | Hesperiidae | 15, 342 bp | KY630504 | Shen et al., (2017) |
| *Ampittia dioscorides* | Hesperiidae | 15, 313 bp | KM102732 | Unpublished |
| *Ampittia virgata* | Hesperiidae | 15, 333 bp | MW288057 | Hao et al., (2021) |
| *Apostictopterus fuliginosus* | Hesperiidae | 15, 417 bp | MH985707 | Han et al., (2018) |
| *Astictopterus jama* | Hesperiidae | 15, 430 bp | MH763663 | Ma et al., (2020) |
| *Barca bicolor* | Hesperiidae | 15, 574 bp | MH985708 | Han et al., (2018) |
| *Burara striata* | Hesperiidae | 15, 327 bp | NC_034676 | Zhang et al., (2017) |
| *Capila translucida* | Hesperiidae | 15, 376 bp | NC_060817 | Xiao et al., (2022) |
| *Carterocephalus silvicola* | Hesperiidae | 15, 765 bp | NC_024646 | Kim et al., (2014) |
| *Celaenorrhinus maculosa* | Hesperiidae | 15, 282 bp | NC_022853 | Wang et al., (2015) |
| *Celaenorrhinus aspersus* | Hesperiidae | 1. 266 bp | NC_060818 | Xiao et al., (2022) |
| *Choaspes benjaminii* | Hesperiidae | 15, 300 bp | NC_024647 | Kim et al., (2014) |
| *Coladenia maeniata* | Hesperiidae | 15, 284 bp | OP723919 | This study |
| *Ctenoptilum vasava* | Hesperiidae | 15, 468 bp | NC_016704 | Cao et al., (2016) |
| *Tagiades tethys* | Hesperiidae | 15, 350 bp | NC_024648 | Zuo et al., (2016) |
| *Tagiades menaka* | Hesperiidae | 15, 294 bp | NC_060823 | Xiao et al., (2022) |
| *Darpa inopinata* | Hesperiidae | 15, 424 bp | OP723920 | This study |
| *Erynnis montanus* | Hesperiidae | 15, 530 bp | NC_021427 | Wang et al., (2014) |
| *Erynnis popoviana* | Hesperiidae | 15, 559 bp | NC_060824 | Xiao et al., (2022) |
| *Euschemon rafflesia* | Hesperiidae | 15, 447 bp | NC_034231 | Zhang et al., (2017) |
| *Erionota torus* | Hesperiidae | 15, 987 bp | MW586888 | unpublished |
| *Gerosis phisara* | Hesperiidae | 15, 429 bp | NC_060819 | Xiao et al., (2022) |
| *Hasora anura* | Hesperiidae | 15, 290 bp | NC_027263 | unpublished |
| *Hasora badra* | Hesperiidae | 15, 324 bp | NC_045249 | unpublished |
| *Halpe nephele* | Hesperiidae | 15, 291 bp | MW288058 | Hao et al., (2021) |
| *Hasora vitta* | Hesperiidae | 15, 282 bp | NC_027170 | Cao et al., (2016) |
| *Heteropterus morpheus* | Hesperiidae | 15, 769 bp | NC_028506 | Unpublished |
| *Isoteinon lamprospilus* | Hesperiidae | 15, 430 bp | MH763664 | Ma et al., (2020) |
| *Lerema accius* | Hesperiidae | 15, 338 bp | NC_029826 | Cong et al., (2016) |
| *Leptalina unicolor* | Hesperiidae | 15, 854 bp | MK265705 | Jeong et al., (2019) |
| *Lobocla bifasciata* | Hesperiidae | 15, 366 bp | KJ629166 | Kim et al., (2014) |
| *Lotongus taprobanus* | Hesperiidae | 15, 191 bp | OP723921 | This study |
| *Matapa aria* | Hesperiidae | 15, 815 bp | OQ784637 | This study |
| *Megathymus beulahae* | Hesperiidae | 15, 412 bp | KY630505 | Zhang et al., (2017) |
| *Megathymus cofaqui* | Hesperiidae | 15, 421 bp | KY630503 | Zhang et al., (2017) |
| *Megathymus streckeri* | Hesperiidae | 15, 507 bp | KY630501 | Zhang et al., (2017) |
| *Megathymus ursus* | Hesperiidae | 15, 396 bp | KY630502 | Zhang et al., (2017) |
| *Megathymus yuccae* | Hesperiidae | 15, 477 bp | KY630500 | Zhang et al., (2017) |
| *Mooreana trichoneura* | Hesperiidae | 15, 232 bp | NC_060820 | Xiao et al., (2022) |
| *Notocrypta curvifascia* | Hesperiidae | 15, 546 bp | MH763665 | Ma et al., (2020) |
| *Onryza maga* | Hesperiidae | 15, 381 bp | MW288059 | Hao et al., (2021) |
| *Ochlodes venata* | Hesperiidae | 15, 622 bp | NC_018048 | Unpublished |
| *Odontoptilum angulatum* | Hesperiidae | 15, 361 bp | MW381783 | Liu et al., (2021) |
| *Parnara guttatus* | Hesperiidae | 15, 441 bp | NC_029136 | Shao et al., (2015) |
| *Potanthus flavus* | Hesperiidae | 15, 267 bp | NC_024650 | Kim et al., (2014) |
| *Pseudocoladenia dea* | Hesperiidae | 15, 374 bp | OP723922 | This study |
| *Pseudocoladenia dan fabia* | Hesperiidae | 15, 358 bp | OP723923 | This study |
| *Pseudocoladenia festa* | Hesperiidae | 15, 382 bp | OP723924 | This study |
| *Pyrgus maculatus* | Hesperiidae | 15, 346 bp | NC_030192 | Unpublished |
| *Satarupa nymphalis* | Hesperiidae | 15, 359 bp | NC_060822 | Xiao et al., (2022) |
| *Signeta flammeata* | Hesperiidae | 14, 917 bp | OQ784638 | This study |
| *Tagiades vajuna* | Hesperiidae | 15, 359 bp | KX865091 | Liu et al., (2017) |
| *Tagiades japetus* | Hesperiidae | 15, 336 bp | OP723925 | This study |
| *Trapezites iacchus* | Hesperiidae | 15, 853 bp | OP723926 | This study |
| *Trapezites symmomus* | Hesperiidae | 15, 387 bp | OQ784639 | This study |
| *Apocheima cinerarium* | Geometridae | 15, 722 bp | NC_024824 | Liu et al., (2014) |
| *Biston suppressaria* | Geometridae | 15, 628 bp | NC_027111 | Chen et al., (2015) |
| *Phthonandria atrilineata* | Geometridae | 15, 499 bp | NC_010522 | Yang et al., (2009) |
| *Graphium timur* | Papilionidae | 15, 226 bp | NC_024098 | Chen et al., (2014) |
| *Papilio glaucus* | Papilionidae | 15, 306 bp | NC_027252 | Shen et al., (2015) |
| *Parnassius apollo* | Papilionidae | 15, 404 bp | NC_024727 | Chen et al., (2014) |

**Supplementary Table S2.** The organization of mitochondrial genomes of nine species. In the column intergenic length, the positive number indicates interval base pairs between genes, while the negative number indicates the overlapping base pairs between genes.

| **species** | ***Abraximorpha esta*** | | | | | | | |
| --- | --- | --- | --- | --- | --- | --- | --- | --- |
| **Gene** | **Start** | **Stop** | **Strand** | **Length** | **start codon** | **stop codon** | **anticodon** | **Intergenic nucleotide** |
| **trnM(atg)** | 1 | 67 | + | 67 |  |  | CAT | 0 |
| **trnI(atc)** | 68 | 137 | + | 70 |  |  | GAT | -3 |
| **trnQ(caa)** | 135 | 203 | - | 69 |  |  | TTG | 91 |
| **ND2** | 295 | 1308 | + | 1014 | ATT | TAA |  | -2 |
| **trnW(tga)** | 1307 | 1373 | + | 67 |  |  | TCA | -8 |
| **trnC(tgc)** | 1366 | 1430 | - | 65 |  |  | GCA | 8 |
| **trnY(tac)** | 1439 | 1504 | - | 66 |  |  | GTA | 9 |
| **COI** | 1514 | 3044 | + | 1531 | CGA | T-- |  | 0 |
| **trnL2(tta)** | 3045 | 3111 | + | 67 |  |  | TAA | 0 |
| **COII** | 3112 | 3787 | + | 660 | ATG | T-- |  | 3 |
| **trnK(aag)** | 3785 | 3855 | + | 71 |  |  | CTT | 15 |
| **trnD(gac)** | 3871 | 3942 | + | 72 |  |  | GTC | 0 |
| **ATP8** | 3943 | 4107 | + | 165 | ATT | TAA |  | -7 |
| **ATP6** | 4101 | 4778 | + | 678 | ATG | TAA |  | -1 |
| **COIII** | 4778 | 5563 | + | 786 | ATG | TAA |  | 2 |
| **trnG(gga)** | 5566 | 5632 | + | 66 |  |  | TCC | 1 |
| **ND3** | 5633 | 5986 | + | 354 | ATT | TAA |  | 21 |
| **trnA(gca)** | 6008 | 6071 | + | 64 |  |  | TGC | 0 |
| **trnR(cga)** | 6072 | 6138 | + | 67 |  |  | TCG | 0 |
| **trnN(aac)** | 6139 | 6205 | + | 67 |  |  | GTT | 95 |
| **trnS2(agc)** | 6301 | 6362 | + | 62 |  |  | GCT | 5 |
| **trnE(gaa)** | 6368 | 6437 | + | 70 |  |  | TTC | 4 |
| **trnF(ttc)** | 6442 | 6509 | - | 68 |  |  | GAA | 0 |
| **ND5** | 6510 | 8253 | - | 1750 | ATT | T-- |  | -3 |
| **trnH(cac)** | 8257 | 8329 | - | 73 |  |  | GTG | 0 |
| **ND4** | 8330 | 9668 | - | 1339 | ATG | T-- |  | -1 |
| **ND4L** | 9668 | 9949 | - | 282 | ATG | TAA |  | 11 |
| **trnT(aca)** | 9961 | 10025 | + | 65 |  |  | TGT | 0 |
| **trnP(cca)** | 10026 | 10090 | - | 65 |  |  | TGG | 2 |
| **ND6** | 10093 | 10633 | + | 541 | ATT | T-- |  | -1 |
| **CYTB** | 10633 | 11784 | + | 1152 | ATG | TAA |  | 0 |
| **trnS1(tca)** | 11785 | 11849 | + | 65 |  |  | TGA | 17 |
| **ND1** | 11867 | 12805 | - | 939 | ATG | TAA |  | 1 |
| **trnL1(cta)** | 12807 | 12874 | - | 68 |  |  | TAG | 0 |
| **lrRNA** | 12875 | 14233 | - | 1359 |  |  |  | 0 |
| **trnV(gta)** | 14234 | 14299 | - | 66 |  |  | TAC | -1 |
| **srRNA** | 14299 | 15069 | - | 771 |  |  |  | 0 |
| **AT-rich region** | 15070 | 15347 |  | 278 |  |  |  | 0 |

| **species** | ***Coladenia maeniata*** | | | | | | | |
| --- | --- | --- | --- | --- | --- | --- | --- | --- |
| **Gene** | **Start** | **Stop** | **Strand** | **Length** | **start codon** | **stop codon** | **anticodon** | **Intergenic nucleotide** |
| **trnM(atg)** | 1 | 68 | + | 68 |  |  | CAT | 0 |
| **trnI(atc)** | 69 | 138 | + | 70 |  |  | GAT | -3 |
| **trnQ(caa)** | 136 | 204 | - | 69 |  |  | TTG | 64 |
| **ND2** | 269 | 1282 | + | 1014 | ATT | TAA |  | -1 |
| **trnW(tga)** | 1282 | 1347 | + | 66 |  |  | TCA | -8 |
| **trnC(tgc)** | 1340 | 1403 | - | 64 |  |  | GCA | 0 |
| **trnY(tac)** | 1404 | 1469 | - | 66 |  |  | GTA | 2 |
| **COI** | 1472 | 3002 | + | 1531 | CGA | T-- |  | 0 |
| **trnL2(tta)** | 3003 | 3069 | + | 67 |  |  | TAA | 0 |
| **COII** | 3070 | 3745 | + | 676 | ATG | T-- |  | -3 |
| **trnK(aag)** | 3743 | 3813 | + | 71 |  |  | CTT | 48 |
| **trnD(gac)** | 3862 | 3930 | + | 69 |  |  | GTC | 0 |
| **ATP8** | 3931 | 4095 | + | 165 | ATT | TAA |  | -7 |
| **ATP6** | 4089 | 4766 | + | 678 | ATG | TAA |  | -1 |
| **COIII** | 4766 | 5551 | + | 786 | ATG | TAA |  | 2 |
| **trnG(gga)** | 5554 | 5618 | + | 65 |  |  | TCC | 0 |
| **ND3** | 5619 | 5972 | + | 354 | ATT | TAA |  | 10 |
| **trnA(gca)** | 5983 | 6050 | + | 68 |  |  | TGC | -1 |
| **trnR(cga)** | 6050 | 6111 | + | 62 |  |  | TCG | 5 |
| **trnN(aac)** | 6117 | 6182 | + | 66 |  |  | GTT | 5 |
| **trnS2(agc)** | 6188 | 6249 | + | 62 |  |  | GCT | 0 |
| **trnE(gaa)** | 6250 | 6324 | + | 75 |  |  | TTC | 0 |
| **trnF(ttc)** | 6325 | 6391 | - | 67 |  |  | GAA | 0 |
| **ND5** | 6392 | 8138 | - | 1747 | ATT | T-- |  | -3 |
| **trnH(cac)** | 8136 | 8202 | - | 67 |  |  | GTG | 0 |
| **ND4** | 8203 | 9541 | - | 1339 | ATG | T-- |  | -1 |
| **ND4L** | 9541 | 9822 | - | 282 | ATG | TAA |  | 5 |
| **trnT(aca)** | 9828 | 9891 | + | 64 |  |  | TGT | 0 |
| **trnP(cca)** | 9892 | 9956 | - | 65 |  |  | TGG | 2 |
| **ND6** | 9959 | 10489 | + | 531 | ATT | TAA |  | -1 |
| **CYTB** | 10489 | 11640 | + | 1152 | ATG |  |  | 2 |
| **trnS1(tca)** | 11643 | 11707 | + | 65 |  |  | TGA | 8 |
| **ND1** | 11716 | 12661 | - | 946 | ATG | T-- |  | 1 |
| **trnL1(cta)** | 12663 | 12732 | - | 70 |  |  | TAG | 0 |
| **lrRNA** | 12733 | 14080 | - | 1348 |  |  |  | 0 |
| **trnV(gta)** | 14081 | 14149 | - | 69 |  |  | TAC | 0 |
| **srRNA** | 14150 | 14926 | - | 777 |  |  |  | 0 |
| **AT-rich region** | 14927 | 15284 |  | 358 |  |  |  |  |
| **species** | ***Darpa inopinata*** | | | | | | | |
| **Gene** | **Start** | **Stop** | **Strand** | **Length** | **start codon** | **stop codon** | **anticodon** | **Intergenic nucleotide** |
| **trnM(atg)** | 1 | 65 | + | 65 |  |  | CAT | 3 |
| **trnI(atc)** | 69 | 133 | + | 65 |  |  | GAT | 2 |
| **trnQ(caa)** | 136 | 205 | - | 70 |  |  | TTG | 92 |
| **ND2** | 298 | 1318 | + | 1021 | ATT | T-- |  | -2 |
| **trnW(tga)** | 1317 | 1383 | + | 67 |  |  | TCA | -8 |
| **trnC(tgc)** | 1376 | 1440 | - | 65 |  |  | GCA | 13 |
| **trnY(tac)** | 1454 | 1519 | - | 66 |  |  | GTA | 7 |
| **COI** | 1527 | 3057 | + | 1531 | CGA | T-- |  | 0 |
| **trnL2(tta)** | 3058 | 3124 | + | 67 |  |  | TAA | 0 |
| **COII** | 3125 | 3803 | + | 679 | ATG | T-- |  | -3 |
| **trnK(aag)** | 3801 | 3871 | + | 71 |  |  | CTT | 27 |
| **trnD(gac)** | 3899 | 3966 | + | 68 |  |  | GTC | 0 |
| **ATP8** | 3967 | 4131 | + | 165 | ATT | TAA |  | -4 |
| **ATP6** | 4128 | 4802 | + | 675 | ATA | TAA |  | -1 |
| **COIII** | 4802 | 5587 | + | 786 | ATG | TAA |  | 2 |
| **trnG(gga)** | 5590 | 5655 | + | 66 |  |  | TCC | 0 |
| **ND3** | 5659 | 6009 | + | 354 | ATT | TAA |  | 3 |
| **trnA(gca)** | 6013 | 6077 | + | 65 |  |  | TGC | -1 |
| **trnR(cga)** | 6077 | 6141 | + | 65 |  |  | TCG | 6 |
| **trnN(aac)** | 6148 | 6212 | + | 65 |  |  | GTT | 3 |
| **trnS2(agc)** | 6216 | 6276 | + | 61 |  |  | GCT | 108 |
| **trnE(gaa)** | 6385 | 6457 | + | 73 |  |  | TTC | 1 |
| **trnF(ttc)** | 6459 | 6521 | - | 63 |  |  | GAA | 3 |
| **ND5** | 6525 | 8276 | - | 1752 | ATT | TAA |  | -14 |
| **trnH(cac)** | 8263 | 8327 | - | 65 |  |  | GTG | 0 |
| **ND4** | 8328 | 9666 | - | 1339 | ATG | T-- |  | 0 |
| **ND4L** | 9667 | 9948 | - | 282 | ATG | TAA |  | 9 |
| **trnT(aca)** | 9958 | 10021 | + | 64 |  |  | TGT | 0 |
| **trnP(cca)** | 10022 | 10087 | - | 66 |  |  | TGG | 2 |
| **ND6** | 10090 | 10626 | + | 537 | ATT | TAA |  | -1 |
| **CYTB** | 10626 | 11774 | + | 1149 | ATG | TAA |  | 6 |
| **trnS1(tca)** | 11781 | 11845 | + | 65 |  |  | TGA | 14 |
| **ND1** | 11860 | 12804 | - | 945 | ATG | TAA |  | 1 |
| **trnL1(cta)** | 12806 | 12874 | - | 69 |  |  | TAG | -1 |
| **lrRNA** | 12874 | 14234 | - | 1361 |  |  |  | 0 |
| **trnV(gta)** | 14235 | 14299 | - | 64 |  |  | TAC | -2 |
| **srRNA** | 14297 | 15073 | - | 777 |  |  |  | 0 |
| **AT-rich region** | 15074 | 15424 |  | 351 |  |  |  | 0 |
| **species** | ***Pseudocoladenia festa*** | | | | | | | |
| **Gene** | **Start** | **Stop** | **Strand** | **Length** | **start codon** | **stop codon** | **anticodon** | **Intergenic nucleotide** |
| **trnM(atg)** | 1 | 67 | + | 67 |  |  | CAT | 4 |
| **trnI(atc)** | 72 | 137 | + | 66 |  |  | GAT | 1 |
| **trnQ(caa)** | 139 | 207 | - | 69 |  |  | TTG | 73 |
| **ND2** | 281 | 1294 | + | 1014 | ATT | TAA |  | -2 |
| **trnW(tga)** | 1293 | 1360 | + | 68 |  |  | TCA | -8 |
| **trnC(tgc)** | 1353 | 1420 | - | 68 |  |  | GCA | 3 |
| **trnY(tac)** | 1424 | 1488 | - | 65 |  |  | GTA | 24 |
| **COI** | 1513 | 3043 | + | 1531 | CGA | T-- |  | 0 |
| **trnL2(tta)** | 3044 | 3110 | + | 67 |  |  | TAA | 0 |
| **COII** | 3111 | 3786 | + | 676 | ATG | T-- |  | 0 |
| **trnK(aag)** | 3787 | 3857 | + | 71 |  |  | CTT | 15 |
| **trnD(gac)** | 3873 | 3939 | + | 67 |  |  | GTC | 0 |
| **ATP8** | 3940 | 4101 | + | 162 | ATT | TAG |  | -7 |
| **ATP6** | 4095 | 4772 | + | 678 | ATG | TAA |  | -1 |
| **COIII** | 4772 | 5557 | + | 786 | ATG | TAA |  | 3 |
| **trnG(gga)** | 5561 | 5625 | + | 65 |  |  | TCC | 0 |
| **ND3** | 5626 | 5979 | + | 339 | ATT | TAA |  | 7 |
| **trnA(gca)** | 5987 | 6053 | + | 67 |  |  | TGC | -1 |
| **trnR(cga)** | 6053 | 6119 | + | 67 |  |  | TCG | 0 |
| **trnN(aac)** | 6120 | 6186 | + | 67 |  |  | GTT | 1 |
| **trnS2(agc)** | 6188 | 6247 | + | 60 |  |  | GCT | 0 |
| **trnE(gaa)** | 6248 | 6311 | + | 64 |  |  | TTC | 0 |
| **trnF(ttc)** | 6312 | 6376 | - | 65 |  |  | GAA | 3 |
| **ND5** | 6380 | 8120 | - | 1741 | ATT | T-- |  | 0 |
| **trnH(cac)** | 8121 | 8193 | - | 73 |  |  | GTG | -5 |
| **ND4** | 8189 | 9518 | - | 1330 | ATG | T-- |  | 4 |
| **ND4L** | 9523 | 9807 | - | 285 | ATG | TAG |  | 2 |
| **trnT(aca)** | 9810 | 9874 | + | 65 |  |  | TGT | 0 |
| **trnP(cca)** | 9875 | 9939 | - | 65 |  |  | TGG | 2 |
| **ND6** | 9942 | 10469 | + | 528 | ATT | TAA |  | -1 |
| **CYTB** | 10469 | 11620 | + | 1152 | ATG | TAA |  | -2 |
| **trnS1(tca)** | 11619 | 11683 | + | 65 |  |  | TGA | 17 |
| **ND1** | 11701 | 12639 | - | 939 | ATG | TAA |  | 3 |
| **trnL1(cta)** | 12643 | 12709 | - | 67 |  |  | TAG | 0 |
| **lrRNA** | 12710 | 14064 | - | 1355 |  |  |  | 0 |
| **trnV(gta)** | 14065 | 14130 | - | 66 |  |  | TAC | 0 |
| **srRNA** | 14131 | 14905 | - | 775 |  |  |  | 0 |
| **AT-rich region** | 14906 | 15382 |  | 477 |  |  |  |  |
| **species** | ***Pseudocoladenia dan fabia*** | | | | | | | |
| **Gene** | **Start** | **Stop** | **Strand** | **Length** | **start codon** | **stop codon** | **anticodon** | **Intergenic nucleotide** |
| **trnM(atg)** | 1 | 69 | + | 69 |  |  | CAT | 0 |
| **trnI(atc)** | 71 | 135 | + | 65 |  |  | GAT | 12 |
| **trnQ(caa)** | 148 | 216 | - | 69 |  |  | TTG | 62 |
| **ND2** | 279 | 1292 | + | 1014 | ATT | TAA |  | -2 |
| **trnW(tga)** | 1291 | 1358 | + | 68 |  |  | TCA | -8 |
| **trnC(tgc)** | 1351 | 1419 | - | 69 |  |  | GCA | 3 |
| **trnY(tac)** | 1423 | 1487 | - | 65 |  |  | GTA | 14 |
| **COI** | 1502 | 3032 | + | 1531 | CGA | T-- |  | 0 |
| **trnL2(tta)** | 3033 | 3099 | + | 67 |  |  | TAA | 0 |
| **COII** | 3100 | 3775 | + | 676 | ATG | T-- |  | 0 |
| **trnK(aag)** | 3776 | 3846 | + | 71 |  |  | CTT | 2 |
| **trnD(gac)** | 3849 | 3915 | + | 67 |  |  | GTC | 0 |
| **ATP8** | 3916 | 4074 | + | 159 | ATT | TAA |  | -7 |
| **ATP6** | 4068 | 4745 | + | 669 | ATG | TAA |  | -1 |
| **COIII** | 4745 | 5530 | + | 786 | ATG | TAA |  | 2 |
| **trnG(gga)** | 5533 | 5597 | + | 65 |  |  | TCC | 0 |
| **ND3** | 5598 | 5951 | + | 354 | ATT | TAA |  | 6 |
| **trnA(gca)** | 5958 | 6024 | + | 67 |  |  | TGC | 0 |
| **trnR(cga)** | 6024 | 6090 | + | 67 |  |  | TCG | 0 |
| **trnN(aac)** | 6091 | 6156 | + | 66 |  |  | GTT | 0 |
| **trnS2(agc)** | 6157 | 6216 | + | 60 |  |  | GCT | 0 |
| **trnE(gaa)** | 6217 | 6282 | + | 66 |  |  | TTC | 0 |
| **trnF(ttc)** | 6283 | 6347 | - | 65 |  |  | GAA | 1 |
| **ND5** | 6349 | 8091 | - | 1743 | ATT | TAA |  | -13 |
| **trnH(cac)** | 8079 | 8156 | - | 78 |  |  | GTG | -1 |
| **ND4** | 8156 | 9496 | - | 1341 | ATG | TAA |  | 4 |
| **ND4L** | 9501 | 9785 | - | 285 | ATG | TAA |  | 2 |
| **trnT(aca)** | 9788 | 9851 | + | 64 |  |  | TGT | 0 |
| **trnP(cca)** | 9852 | 9916 | - | 65 |  |  | TGG | 2 |
| **ND6** | 9919 | 10452 | + | 534 | ATT | TAA |  | -7 |
| **CYTB** | 10446 | 11597 | + | 1152 | ATG | TAA |  | -2 |
| **trnS1(tca)** | 11596 | 11661 | + | 66 |  |  | TGA | 17 |
| **ND1** | 11679 | 12617 | - | 939 | ATG | TAA |  | 3 |
| **trnL1(cta)** | 12621 | 12688 | - | 68 |  |  | TAG | 0 |
| **lrRNA** | 12689 | 14048 | - | 1360 |  |  |  | 0 |
| **trnV(gta)** | 14049 | 14115 | - | 67 |  |  | TAC | 0 |
| **srRNA** | 14116 | 14888 | - | 773 |  |  |  | 0 |
| **AT-rich region** | 14889 | 15358 |  | 470 |  |  |  |  |
| **species** | ***Pseudocoladenia dea*** | | | | | | | |
| **Gene** | **Start** | **Stop** | **Strand** | **Length** | **start codon** | **stop codon** | **anticodon** | **Intergenic nucleotide** |
| **trnM(atg)** | 1 | 69 | + | 69 |  |  | CAT | 1 |
| **trnI(atc)** | 71 | 135 | + | 65 |  |  | GAT | 12 |
| **trnQ(caa)** | 148 | 216 | - | 69 |  |  | TTG | 62 |
| **ND2** | 279 | 1292 | + | 1014 | ATT | TAA |  | -2 |
| **trnW(tga)** | 1291 | 1358 | + | 68 |  |  | TCA | -8 |
| **trnC(tgc)** | 1351 | 1419 | - | 69 |  |  | GCA | 3 |
| **trnY(tac)** | 1423 | 1487 | - | 65 |  |  | GTA | 14 |
| **COI** | 1502 | 3032 | + | 1531 | CGA | T-- |  | 0 |
| **trnL2(tta)** | 3033 | 3099 | + | 67 |  |  | TAA | 0 |
| **COII** | 3100 | 3775 | + | 676 | ATG | T-- |  | 0 |
| **trnK(aag)** | 3776 | 3846 | + | 71 |  |  | CTT | 2 |
| **trnD(gac)** | 3849 | 3915 | + | 67 |  |  | GTC | 0 |
| **ATP8** | 3916 | 4074 | + | 159 | ATT | TAA |  | -7 |
| **ATP6** | 4068 | 4745 | + | 678 | ATG | TAA |  | -1 |
| **COIII** | 4745 | 5530 | + | 786 | ATG | TAA |  | 2 |
| **trnG(gga)** | 5533 | 5599 | + | 67 |  |  | TCC | 0 |
| **ND3** | 5600 | 5953 | + | 354 | ATT | TAA |  | 7 |
| **trnA(gca)** | 5961 | 6027 | + | 67 |  |  | TGC | 19 |
| **trnR(cga)** | 6047 | 6112 | + | 66 |  |  | TCG | 0 |
| **trnN(aac)** | 6113 | 6178 | + | 66 |  |  | GTT | 0 |
| **trnS2(agc)** | 6179 | 6238 | + | 60 |  |  | GCT | 0 |
| **trnE(gaa)** | 6239 | 6304 | + | 66 |  |  | TTC | 0 |
| **trnF(ttc)** | 6305 | 6369 | - | 65 |  |  | GAA | 3 |
| **ND5** | 6373 | 8113 | - | 1741 | ATT | T-- |  | -13 |
| **trnH(cac)** | 8101 | 8183 | - | 83 |  |  | GTG | -7 |
| **ND4** | 8177 | 9517 | - | 1341 | ATG | TAA |  | 4 |
| **ND4L** | 9522 | 9806 | - | 285 | ATG | TAA |  | 2 |
| **trnT(aca)** | 9809 | 9872 | + | 64 |  |  | TGT | 0 |
| **trnP(cca)** | 9873 | 9937 | - | 65 |  |  | TGG | 2 |
| **ND6** | 9940 | 10473 | + | 534 | ATT | TAA |  | -7 |
| **CYTB** | 10467 | 11618 | + | 1152 | ATG | TAA |  | -2 |
| **trnS1(tca)** | 11617 | 11682 | + | 66 |  |  | TGA | 17 |
| **ND1** | 11700 | 12641 | - | 942 | ATG | TAA |  | 0 |
| **trnL1(cta)** | 12642 | 12708 | - | 67 |  |  | TAG | 0 |
| **lrRNA** | 12709 | 14070 | - | 1362 |  |  |  | 0 |
| **trnV(gta)** | 14071 | 14137 | - | 67 |  |  | TAC | 0 |
| **srRNA** | 14138 | 14914 | - | 777 |  |  |  | 0 |
| **AT-rich region** | 14915 | 15374 |  | 460 |  |  |  | 0 |
| **species** | ***Trapezites iacchus*** | | | | | | | |
| **Gene** | **Start** | **Stop** | **Strand** | **Length** | **start codon** | **stop codon** | **anticodon** | **Intergenic nucleotide** |
| **trnM(atg)** | 1 | 73 | + | 73 |  |  | CAT | -3 |
| **trnI(atc)** | 71 | 134 | + | 64 |  |  | GAT | -3 |
| **trnQ(caa)** | 132 | 200 | - | 69 |  |  | TTG | 98 |
| **ND2** | 299 | 1312 | + | 1014 | ATT | TAA |  | -2 |
| **trnW(tga)** | 1311 | 1377 | + | 67 |  |  | TCA | -8 |
| **trnC(tgc)** | 1370 | 1440 | - | 71 |  |  | GCA | 26 |
| **trnY(tac)** | 1467 | 1531 | - | 65 |  |  | GTA | 12 |
| **COI** | 1544 | 3074 | + | 1531 | CGA | T-- |  | 0 |
| **trnL2(tta)** | 3075 | 3141 | + | 67 |  |  | TAA | 0 |
| **COII** | 3142 | 3820 | + | 679 | ATG | T-- |  | 0 |
| **trnK(aag)** | 3821 | 3891 | + | 71 |  |  | CTT | 2 |
| **trnD(gac)** | 3894 | 3960 | + | 67 |  |  | GTC | 0 |
| **ATP8** | 3961 | 4119 | + | 159 | ATT | TAA |  | -7 |
| **ATP6** | 4113 | 4787 | + | 675 |  | TAA |  | 23 |
| **COIII** | 4811 | 5596 | + | 786 | ATG | TAA |  | 2 |
| **trnG(gga)** | 5599 | 5666 | + | 68 |  |  | TCC | 0 |
| **ND3** | 5667 | 6020 | + | 354 | ATT | TAA |  | 31 |
| **trnA(gca)** | 6052 | 6122 | + | 71 |  |  | TGC | 67 |
| **trnR(cga)** | 6190 | 6252 | + | 63 |  |  | TCG | 1 |
| **trnN(aac)** | 6254 | 6320 | + | 67 |  |  | GTT | 0 |
| **trnS2(agc)** | 6321 | 6380 | + | 60 |  |  | GCT | 93 |
| **trnE(gaa)** | 6474 | 6542 | + | 69 |  |  | TTC | 201 |
| **trnF(ttc)** | 6744 | 6810 | - | 67 |  |  | GAA | -4 |
| **ND5** | 6807 | 8562 | - | 1756 | ATT | T-- |  | 0 |
| **trnH(cac)** | 8563 | 8640 | - | 78 |  |  | GTG | -7 |
| **ND4** | 8634 | 9974 | - | 1341 | ATG | TAA |  | 3 |
| **ND4L** | 9978 | 10262 | - | 285 | ATG | TAG |  | 11 |
| **trnT(aca)** | 10274 | 10336 | + | 63 |  |  | TGT | 0 |
| **trnP(cca)** | 10337 | 10402 | - | 66 |  |  | TGG | 2 |
| **ND6** | 10405 | 10941 | + | 537 | ATA | TAA |  | -1 |
| **CYTB** | 10941 | 12089 | + | 1149 | ATG | TAA |  | -2 |
| **trnS1(tca)** | 12088 | 12155 | + | 68 |  |  | TGA | 25 |
| **ND1** | 12181 | 13131 | - | 951 | ATA | TAA |  | -6 |
| **trnL1(cta)** | 13126 | 13194 | - | 69 |  |  | TAG | 0 |
| **lrRNA** | 13195 | 14639 | - | 1445 |  |  |  | 7 |
| **trnV(gta)** | 14647 | 14711 | - | 65 |  |  | TAC | 0 |
| **srRNA** | 14712 | 15496 | - | 785 |  |  |  | 0 |
| **AT-rich region** | 15497 | 15853 |  | 357 |  |  |  | 0 |
| **species** | ***Lotongus sarala chinensis*** | | | | | | | |
| **Gene** | **Start** | **Stop** | **Strand** | **Length** | **start codon** | **stop codon** | **anticodon** | **Intergenic nucleotide** |
| **trnM(atg)** | 1 | 68 | + | 68 |  |  | CAT | 3 |
| **trnI(atc)** | 72 | 139 | + | 68 |  |  | GAT | -3 |
| **trnQ(caa)** | 137 | 206 | - | 70 |  |  | TTG | 68 |
| **ND2** | 275 | 1288 | + | 1014 | ATC | TAA |  | 4 |
| **trnW(tga)** | 1293 | 1361 | + | 69 |  |  | TCA | -8 |
| **trnC(tgc)** | 1354 | 1417 | - | 64 |  |  | GCA | 13 |
| **trnY(tac)** | 1431 | 1496 | - | 66 |  |  | GTA | 28 |
| **COI** | 1525 | 3055 | + | 1531 | CGA | T-- |  | 0 |
| **trnL2(tta)** | 3056 | 3122 | + | 67 |  |  | TAA | 1 |
| **COII** | 3124 | 3802 | + | 679 | ATG | T-- |  | 0 |
| **trnK(aag)** | 3803 | 3873 | + | 71 |  |  | CTT | 6 |
| **trnD(gac)** | 3880 | 3946 | + | 67 |  |  | GTC | 0 |
| **ATP8** | 3947 | 4117 | + | 171 | ATC | TAG |  | -7 |
| **ATP6** | 4111 | 4791 | + | 681 | ATG | TAA |  | -1 |
| **COIII** | 4791 | 5576 | + | 786 | ATG | TAA |  | 2 |
| **trnG(gga)** | 5579 | 5643 | + | 65 |  |  | TCC | 0 |
| **ND3** | 5644 | 5997 | + | 354 | ATT | TAA |  | 5 |
| **trnA(gca)** | 6003 | 6071 | + | 69 |  |  | TGC | -1 |
| **trnR(cga)** | 6071 | 6132 | + | 62 |  |  | TCG | 0 |
| **trnN(aac)** | 6133 | 6197 | + | 65 |  |  | GTT | 10 |
| **trnS2(agc)** | 6208 | 6267 | + | 60 |  |  | GCT | 28 |
| **trnE(gaa)** | 6296 | 6363 | + | 68 |  |  | TTC | 0 |
| **trnF(ttc)** | 6364 | 6427 | - | 64 |  |  | GAA | 0 |
| **ND5** | 6428 | 8168 | - | 1741 | ATT | T-- |  | -27 |
| **trnH(cac)** | 8142 | 8232 | - | 91 |  |  | GTG | 0 |
| **ND4** | 8233 | 9571 | - | 1339 | ATG | T-- |  | 5 |
| **ND4L** | 9578 | 9868 | - | 291 | ATA | TAA |  | -8 |
| **trnT(aca)** | 9861 | 9925 | + | 65 |  |  | TGT | 0 |
| **trnP(cca)** | 9926 | 9991 | - | 66 |  |  | TGG | 2 |
| **ND6** | 9994 | 10527 | + | 534 | ATT | TAA |  | 3 |
| **CYTB** | 10531 | 11682 | + | 1152 | ATG | TAA |  | -2 |
| **trnS1(tca)** | 11681 | 11747 | + | 67 |  |  | TGA | 19 |
| **ND1** | 11767 | 12705 | - | 939 | ATA | TAA |  | 0 |
| **trnL1(cta)** | 12706 | 12782 | - | 77 |  |  | TAG | 0 |
| **lrRNA** | 12783 | 14152 | - | 1370 |  |  |  | 1 |
| **trnV(gta)** | 14154 | 14222 | - | 69 |  |  | TAC | 0 |
| **srRNA** | 14223 | 15001 | - | 779 |  |  |  | 0 |
| **AT-rich region** | 15002 | 15853 |  | 852 |  |  |  | 0 |
| **species** | ***Matapa aria*** | | | | | | | |
| **Gene** | **Start** | **Stop** | **Strand** | **Length** | **start codon** | **stop codon** | **anticodon** | **Intergenic nucleotide** |
| **trnM(atg)** | 1 | 69 | + | 69 |  |  | CAT | 1 |
| **trnI(atc)** | 71 | 135 | + | 65 |  |  | GAT | 5 |
| **trnQ(caa)** | 141 | 209 | - | 69 |  |  | TTG | 75 |
| **ND2** | 285 | 1307 | + | 1023 | ATT | TAA |  | -10 |
| **trnW(tga)** | 1298 | 1365 | + | 68 |  |  | TCA | -8 |
| **trnC(tgc)** | 1358 | 1424 | - | 67 |  |  | GCA | 6 |
| **trnY(tac)** | 1431 | 1498 | - | 68 |  |  | GTA | 7 |
| **COI** | 1506 | 3036 | + | 1531 |  |  |  | 3 |
| **trnL2(tta)** | 3040 | 3106 | + | 67 |  |  | TAA | 0 |
| **COII** | 3107 | 3782 | + | 676 | ATG | T-- |  | 0 |
| **trnK(aag)** | 3783 | 3853 | + | 71 |  |  | CTT | 2 |
| **trnD(gac)** | 3856 | 3923 | + | 68 |  |  | GTC | 0 |
| **ATP8** | 3924 | 4088 | + | 165 | ATT | TAA |  | -7 |
| **ATP6** | 4082 | 4756 | + | 675 | ATG | TAA |  | -1 |
| **COIII** | 4756 | 5541 | + | 786 | ATG | TAA |  | 2 |
| **trnG(gga)** | 5544 | 5610 | + | 67 |  |  | TCC | 0 |
| **ND3** | 5611 | 5964 | + | 354 | ATT | TAA |  | 3 |
| **trnA(gca)** | 5968 | 6035 | + | 68 |  |  | TGC | 75 |
| **trnR(cga)** | 6111 | 6172 | + | 62 |  |  | TCG | 0 |
| **trnN(aac)** | 6173 | 6239 | + | 67 |  |  | GTT | 9 |
| **trnS2(agc)** | 6249 | 6310 | + | 62 |  |  | GCT | 56 |
| **trnE(gaa)** | 6367 | 6434 | + | 68 |  |  | TTC | -2 |
| **trnF(ttc)** | 6433 | 6500 | - | 68 |  |  | GAA | 0 |
| **ND5** | 6501 | 8238 | - | 1738 | ATT | T-- |  | 0 |
| **trnH(cac)** | 8239 | 8304 | - | 66 |  |  | GTG | 0 |
| **ND4** | 8305 | 9643 | - | 1339 | ATG | T-- |  | 3 |
| **ND4L** | 9647 | 9955 | - | 309 | ATG | TAA |  | -25 |
| **trnT(aca)** | 9931 | 9995 | + | 65 |  |  | TGT | 0 |
| **trnP(cca)** | 9996 | 10061 | - | 66 |  |  | TGG | 2 |
| **ND6** | 10064 | 10600 | + | 537 | ATA | TAA |  | -1 |
| **CYTB** | 10600 | 11751 | + | 1152 | ATG | TAA |  | -2 |
| **trnS1(tca)** | 11750 | 11814 | + | 65 |  |  | TGA | 18 |
| **ND1** | 11833 | 12771 | - | 939 | ATT | TAA |  | 0 |
| **trnL1(cta)** | 12772 | 12842 | - | 71 |  |  | TAG | 0 |
| **lrRNA** | 12843 | 14252 | - | 1410 |  |  |  | 0 |
| **trnV(gta)** | 14253 | 14325 | - | 73 |  |  | TAC | 0 |
| **srRNA** | 14326 | 15116 | - | 791 |  |  |  | 0 |
| **AT-rich region** | 15117 | 15815 |  | 699 |  |  |  | 0 |

**Supplementary Table S3.** The collecting information of the specimens used in this study. * denotes the four that have been reported on NCBI, but not directly available to us. So we sequenced the mitochondrial data.

| Species | Collecting locality | time |
| --- | --- | --- |
| *Abraximorpha esta* | Baoshan, Yunnan, China | Ⅶ. 2018 |
| *Coladenia maeniata* | Kangding, Sichuan, China | Ⅵ. 2018 |
| *Darpa inopinata* | Yinggeling, Hainan, China | Ⅸ. 2016 |
| *Matapa aria* | Qingyuan County, Zhejiang, China | Ⅷ.2018 |
| *Pseudocoladenia dan fabia* | Yingde, Guangdong, China | Ⅵ. 2017 |
| *Pseudocoladenia dea* | Longcanggou, Sichuan,China | Ⅵ. 2017 |
| *Pseudocoladenia festa* | Moxi, Sichuan, China | Ⅵ. 2018 |
| *Trapezites iacchus* | Moogelah, Queensland, Australia | Ⅰ. 2017 |
| *Lotongus sarala chinensis* | Zhenghe County, Fujian, China | Ⅷ.2018 |
| *Tagiades japentus**** | Ambon, Indonesia | XI.2016 |
| *Trapezites symmonus** | N.S.W.: Mt. Mackinzeli, Australia | 1. 2017 |
| *Signeta flammeata** | N.S.W.: Barrington Tops, Australia | VII.2011 |
| *Lotongus calathus taprobanus** | Sulawesi, Indonesia | XII.2018 |

**
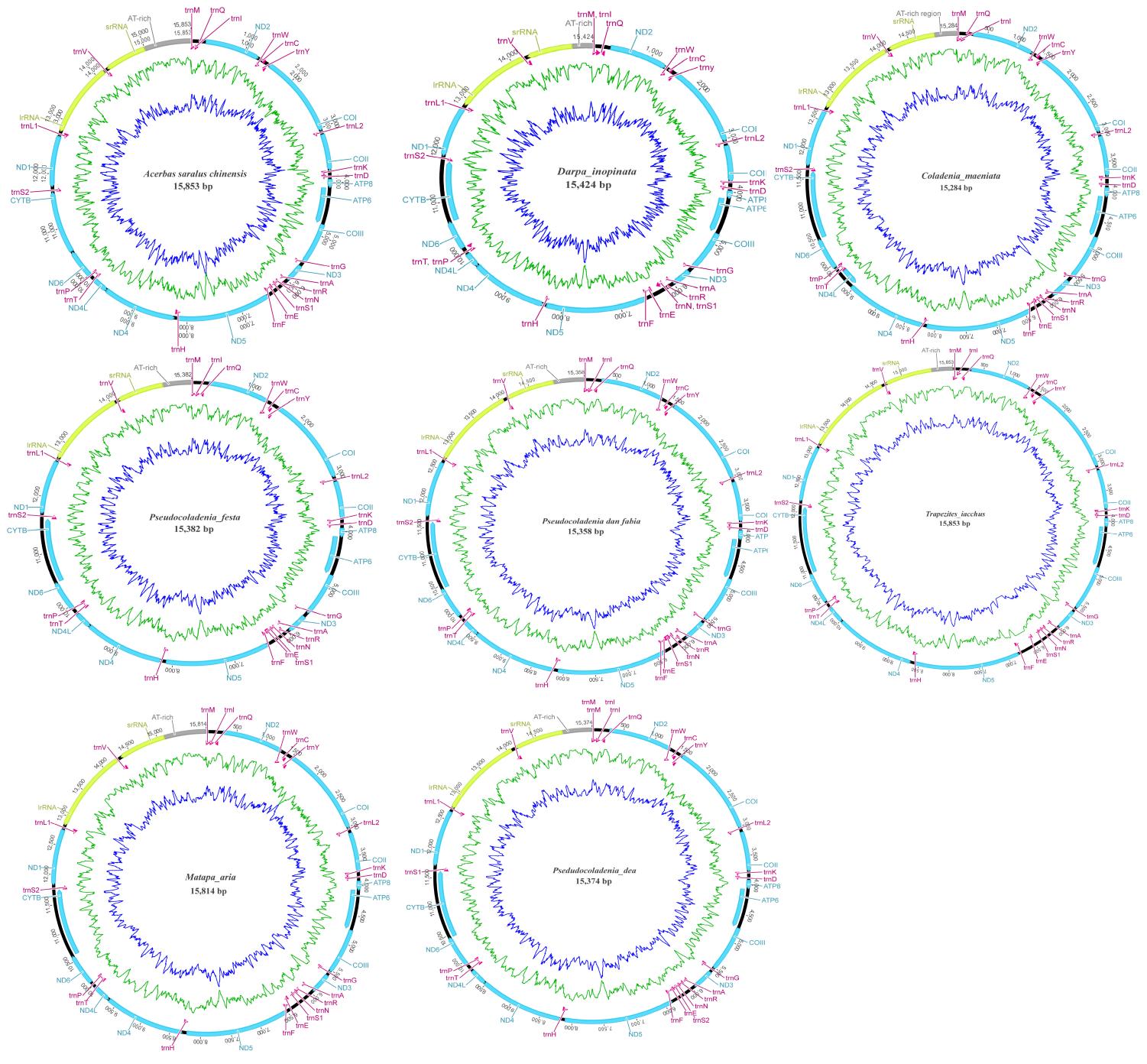
**

**Supplementary Fig. S1.** Mitochondrial genome circle map of 9 species. Use Geneious v7.1.4 software for diagramming.


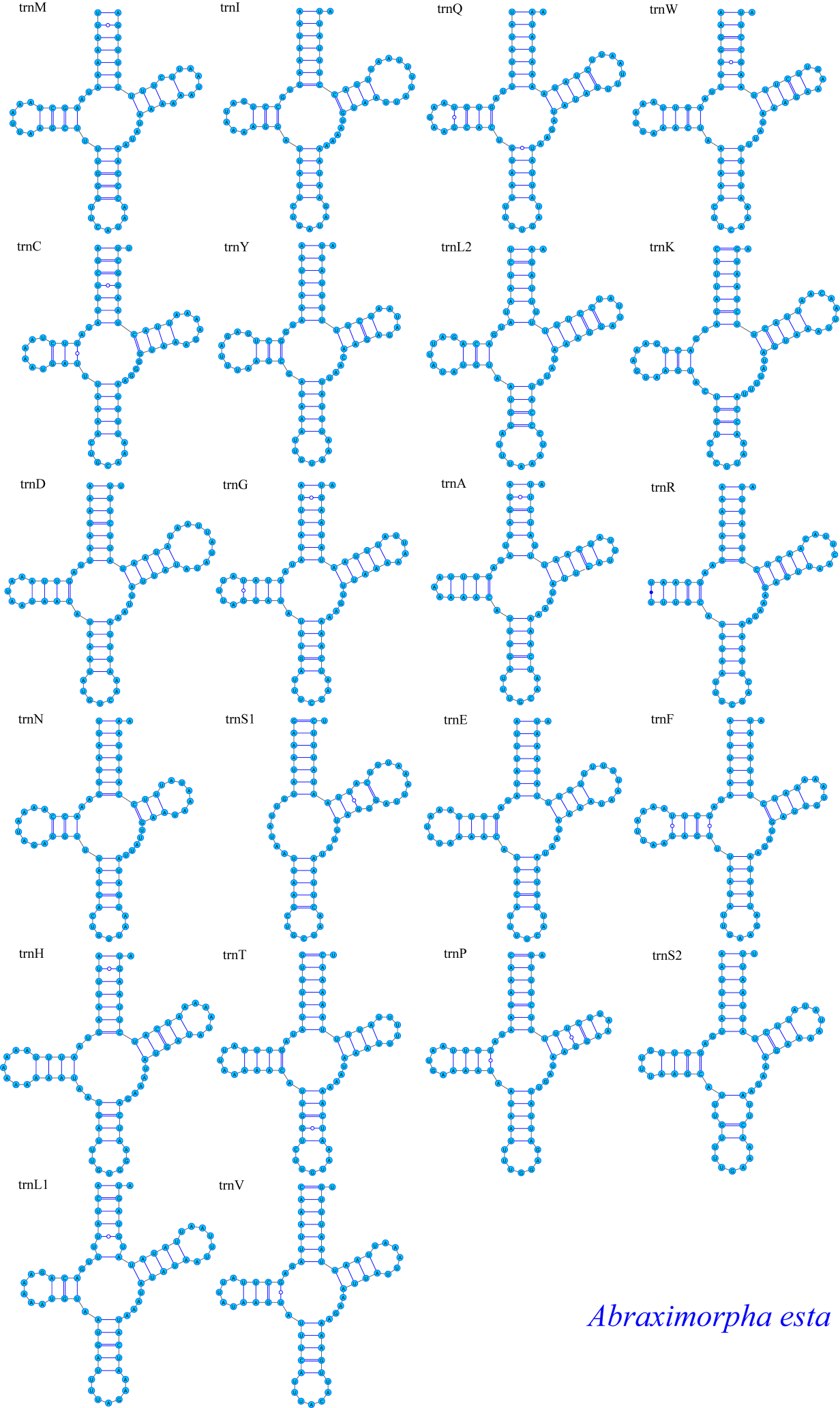


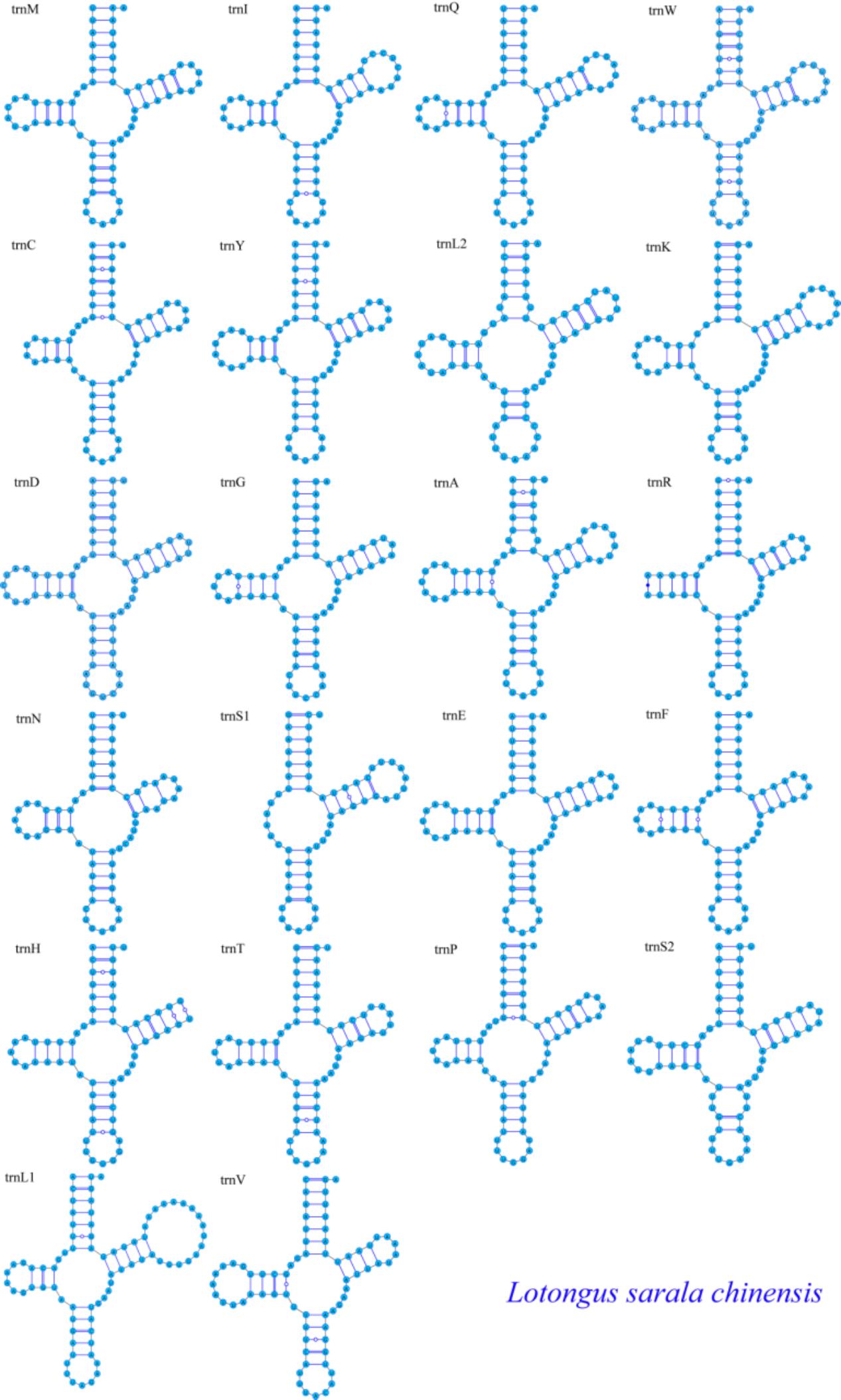


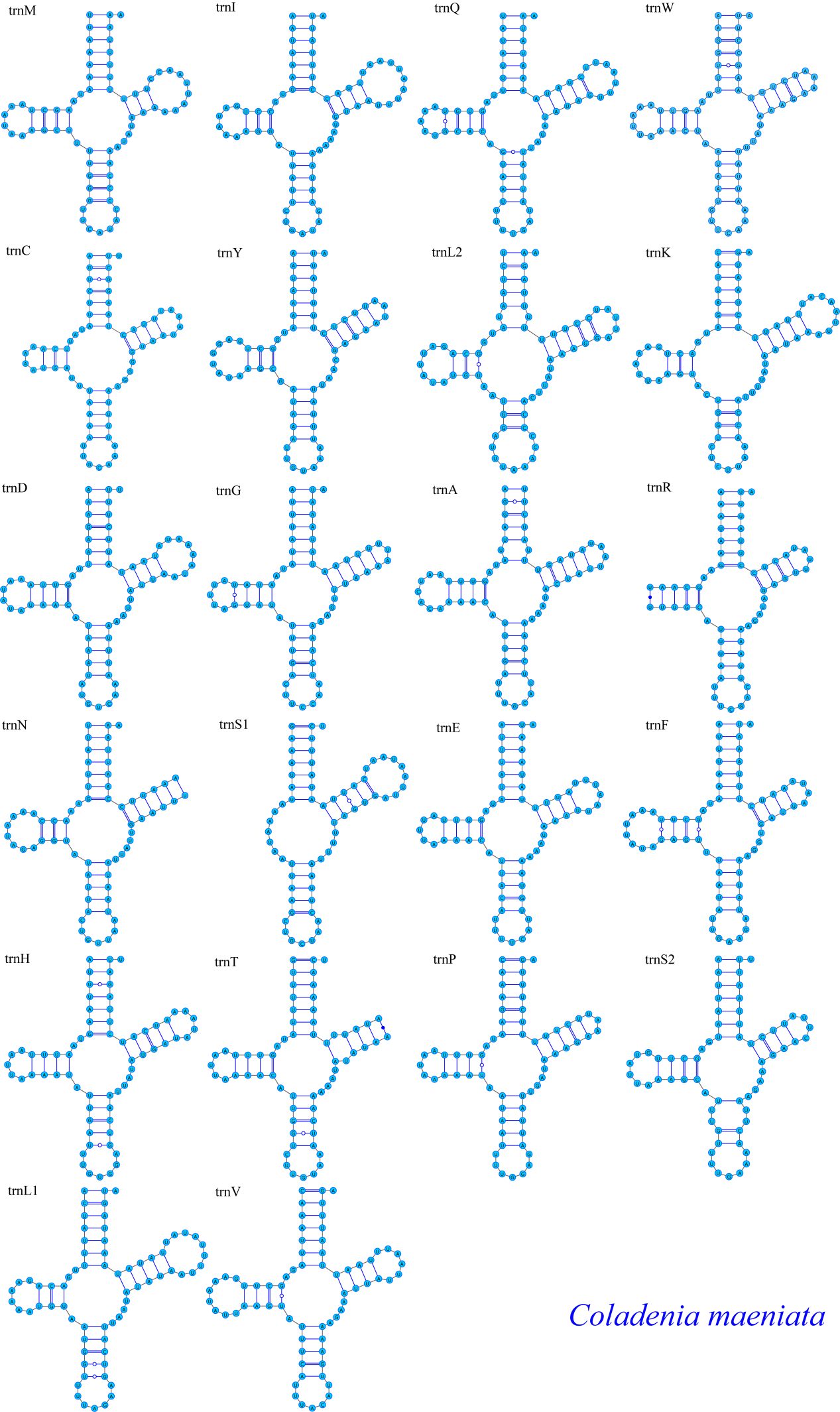


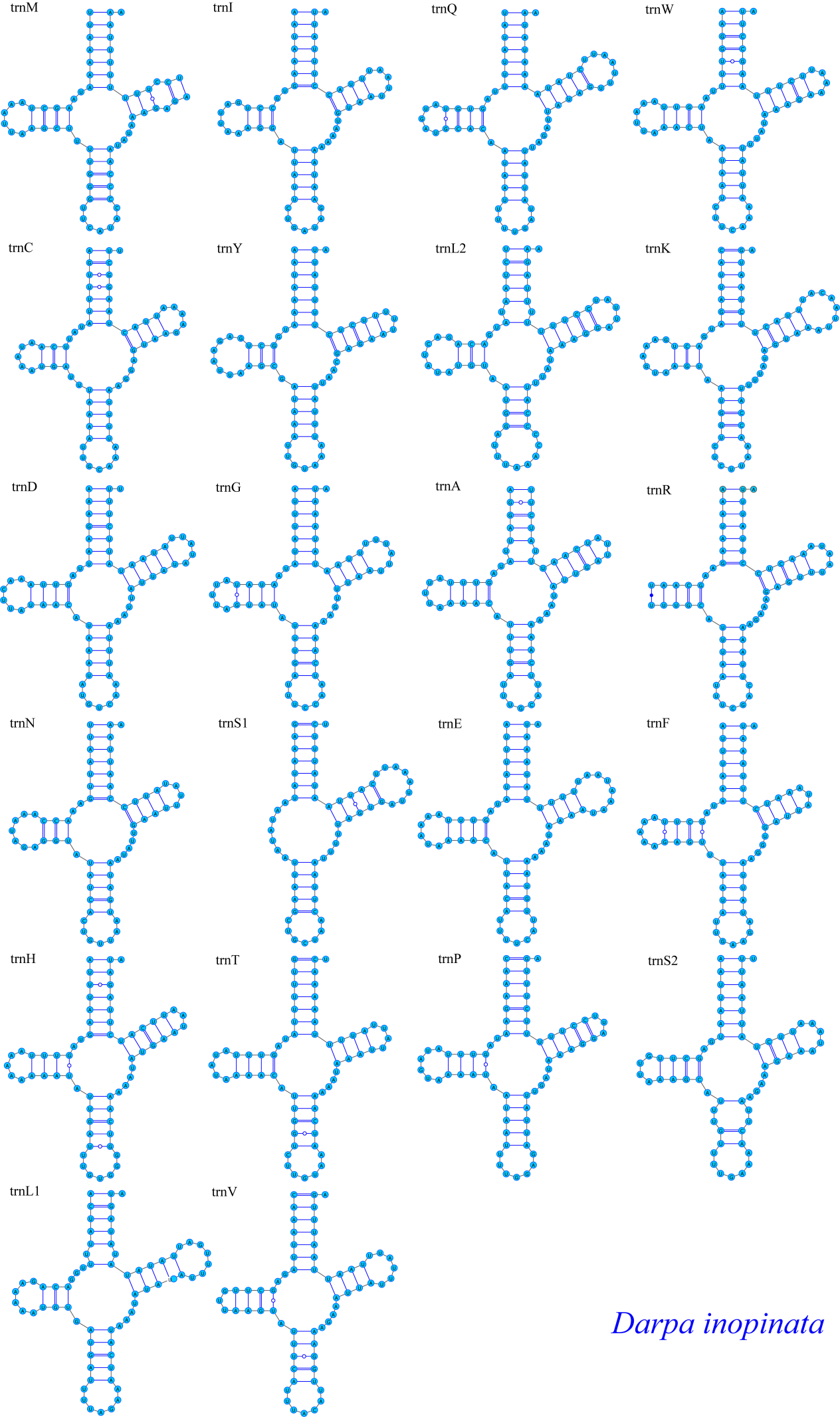


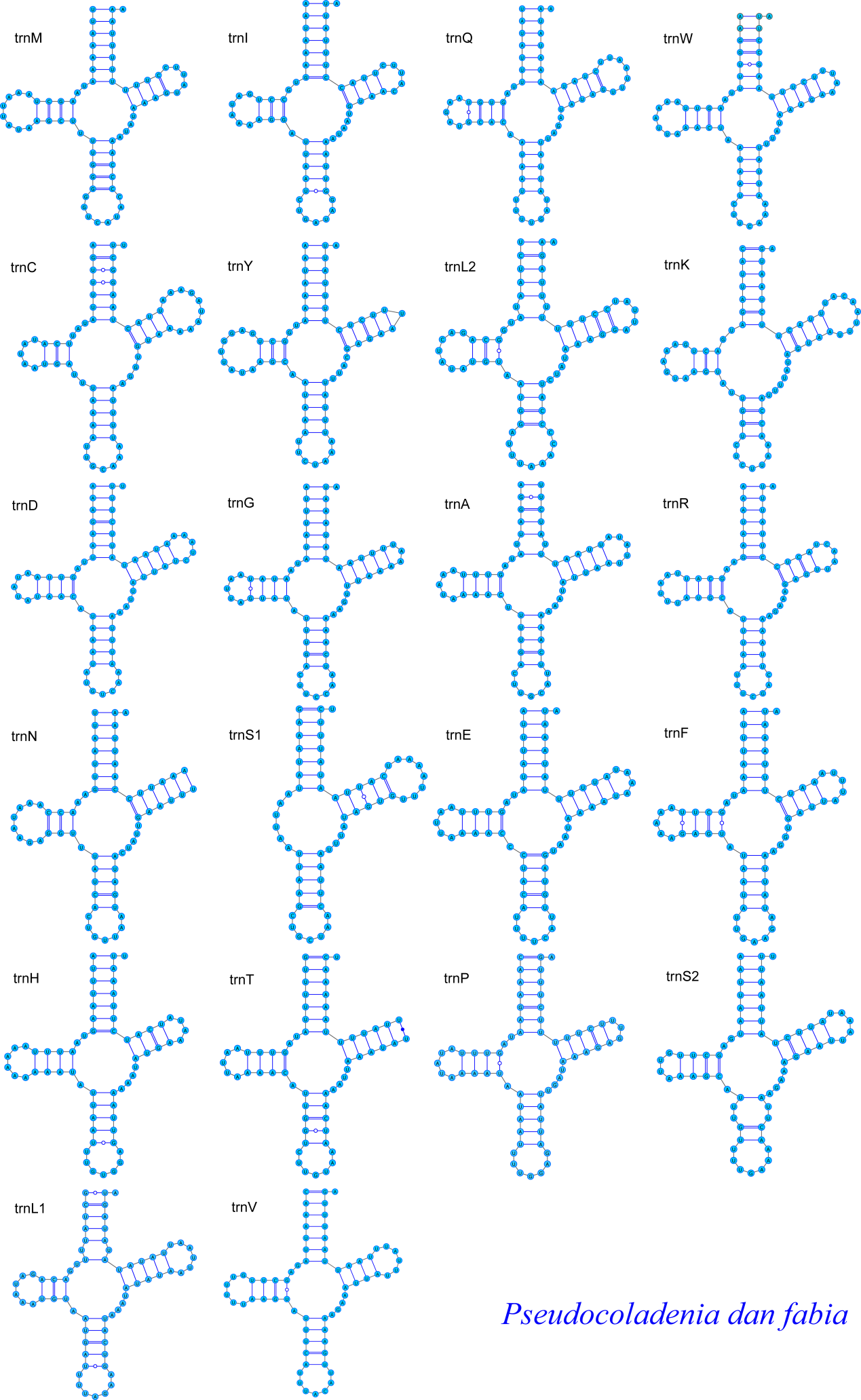


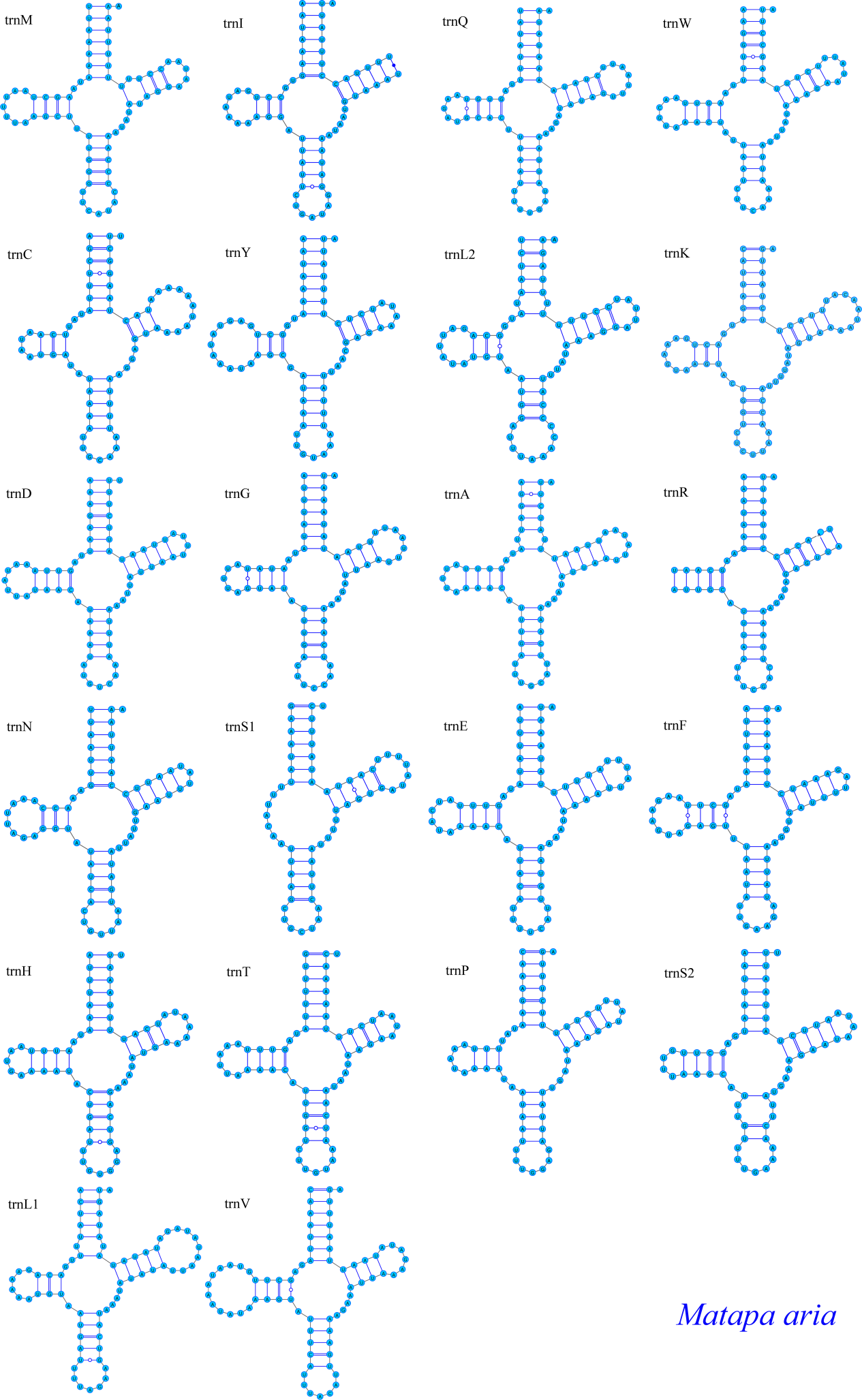


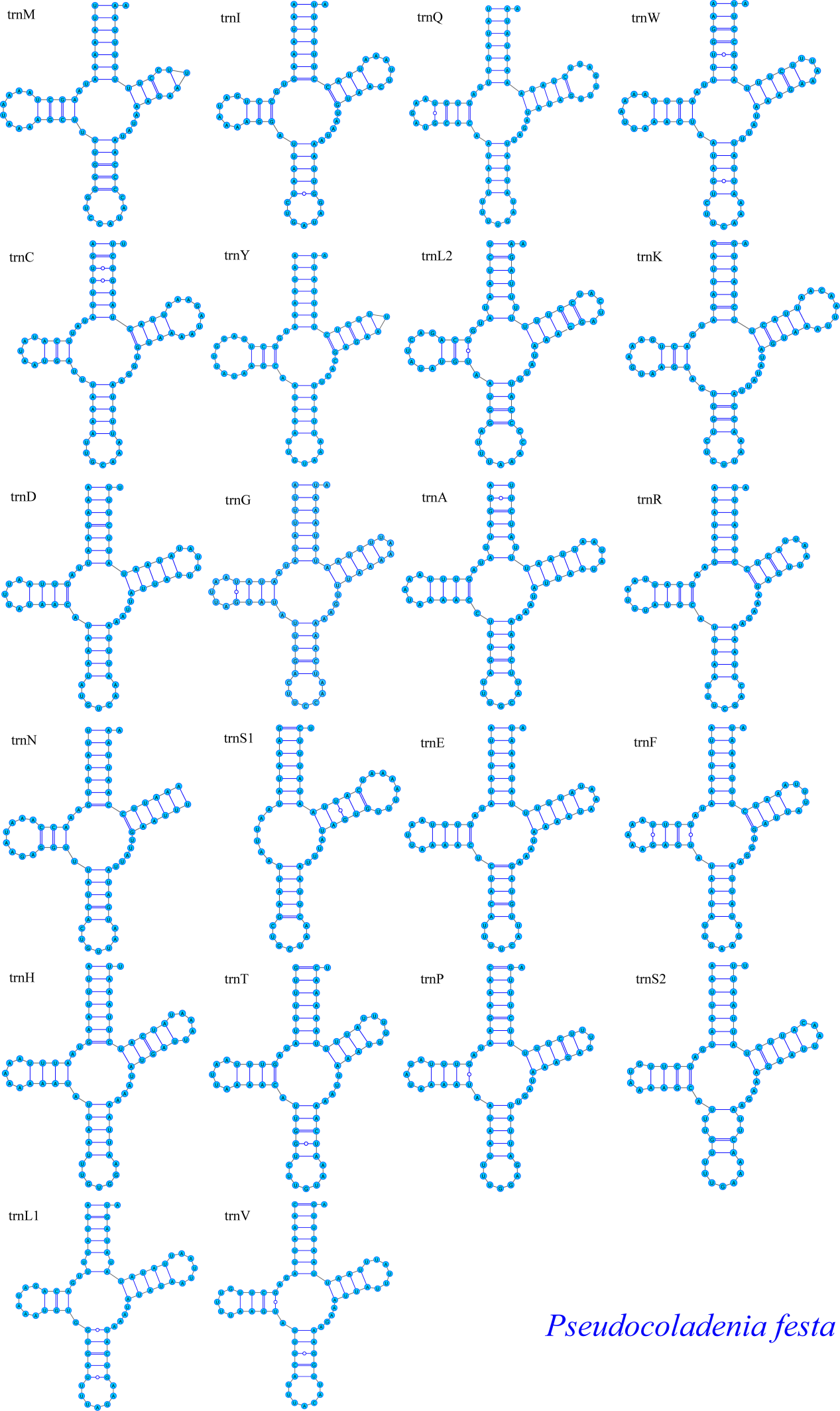


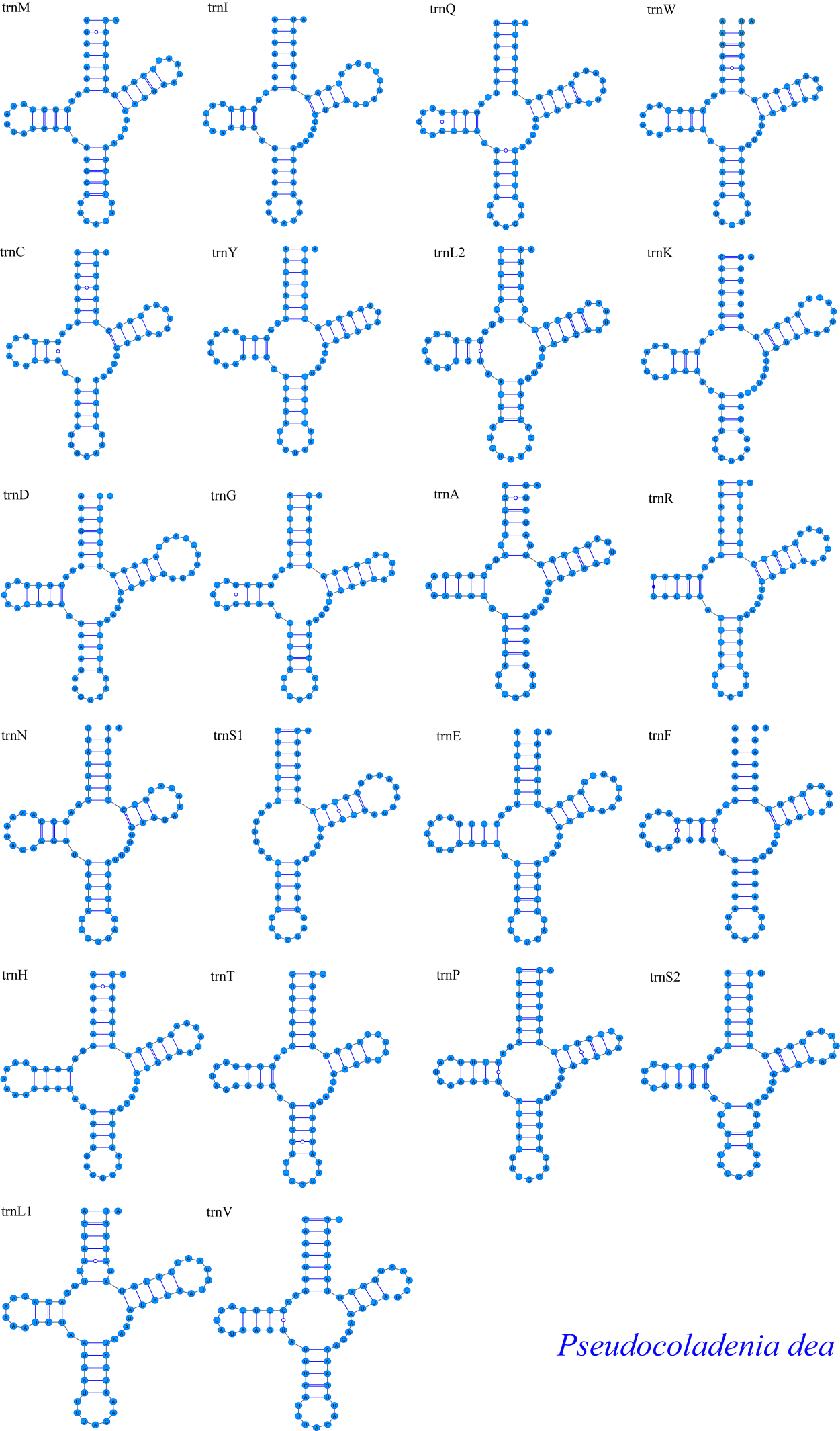


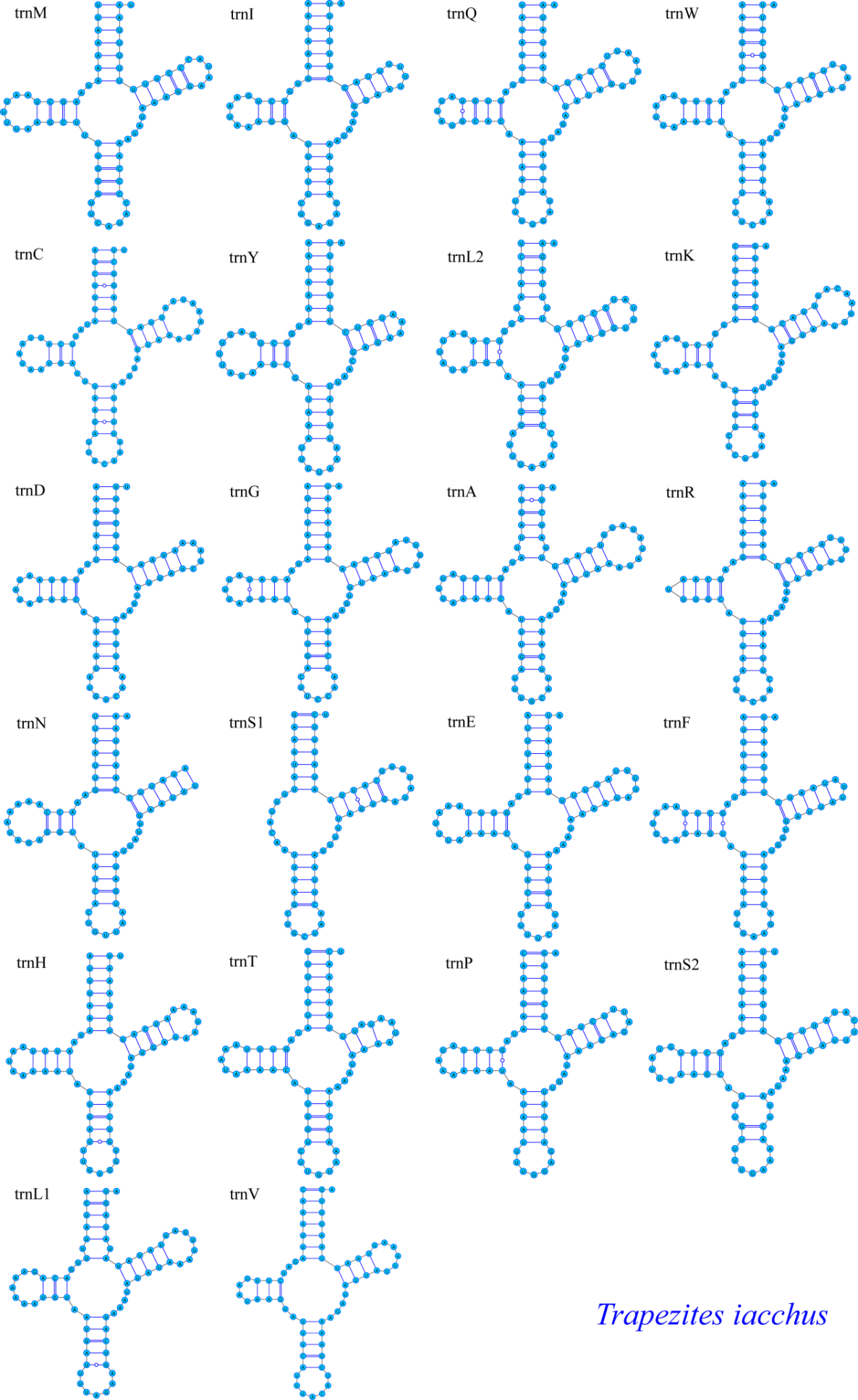


**Supplementary Fig. S2.** Predicted secondary clover-leaf structure for the tRNA genes of nine Hesperiidae species sequenced in this study.

**Supplementary file S1.** PRT data partitioning (a) and PartitionFinder compute the optimal model (b.c).

**a. Data partitioning**

| ND2= 1-1024; | trnM= 10937-11019; | trnN= 11823-11894; |
| --- | --- | --- |
| CO1= 1025-2558; | trn1= 11020-11091; | trnS1= 11895-11961; |
| CO11 = 2559-3240; | trnQ= 11092-11164; | trnE= 11962-12037; |
| ATP8 = 3241-3444; | trnW = 11165-11237; | trnF= 12038-12109; |
| ATP6= 3445-4143; | trnC= 11238-11313; | trnH= 12110-12186; |
| CO111= 4144-4936; | trnY= 11314-11387; | trnT= 12187-12254; |
| ND3= 4937-5290; | trnL2= 11388-11456; | trnP= 12255-12322; |
| ND5= 5291-7051; | trnK= 11457-11527; | trnS2= 12323-12395; |
| ND4= 7052-7976; | trnD= 11528-11604; | trnL1 = 12396-12473; |
| ND4L = 7977-8267; | trnG= 11605-11676; | trnV = 12474-12549; |
| ND6= 8268-8835; | trnA= 11677-11749; | lrRNA= 12550-14178; |
| CYTB = 8836-9990; | trnR= 11750-11822; | srRNA= 14179-15066; |
| ND1= 9991-10936; |  |  |

**b. ML tree optimal model**

#nexus

begin sets;

charset Subset1 = 1-1024\3;

charset Subset2 = 2-1024\3;

charset Subset3 = 3-1024\3;

charset Subset4 = 1025-2558\3;

charset Subset5 = 1026-2558\3;

charset Subset6 = 4144-4936\3 1027-2558\3;

charset Subset7 = 11458-11527\3 2559-3240\3;

charset Subset8 = 8837-9990\3 2560-3240\3;

charset Subset9 = 2561-3240\3;

charset Subset10 = 3241-3444\3;

charset Subset11 = 3242-3444\3 12040-12109\3;

charset Subset12 = 5292-7051\3 3243-3444\3;

charset Subset13 = 3445-4143\3;

charset Subset14 = 3446-4143\3;

charset Subset15 = 3447-4143\3 9991-10936\3;

charset Subset16 = 4145-4936\3 11459-11527\3;

charset Subset17 = 4146-4936\3;

charset Subset18 = 11092-11164\3 11390-11456\3 4937-5290\3 11166-11237\3 11093-11164\3;

charset Subset19 = 4938-5290\3;

charset Subset20 = 4939-5290\3 8838-9990\3;

charset Subset21 = 12476-12549\3 5291-7051\3;

charset Subset22 = 5293-7051\3;

charset Subset23 = 7052-7976\3 11314-11387\3;

charset Subset24 = 7053-7976\3;

charset Subset25 = 7054-7976\3 14179-15066\3;

charset Subset26 = 9992-10936\3 7977-8267\3;

charset Subset27 = 7978-8267\3 10939-11019\3;

charset Subset28 = 11240-11313\3 7979-8267\3;

charset Subset29 = 12039-12109\3 11962-12037\3 8268-8835\3;

charset Subset30 = 8269-8835\3;

charset Subset31 = 8270-8835\3;

charset Subset32 = 8836-9990\3;

charset Subset33 = 9993-10936\3;

charset Subset34 = 10937-11019\3 11528-11604\3 11752-11822\3;

charset Subset35 = 11388-11456\3 10938-11019\3;

charset Subset36 = 11751-11822\3 11020-11091\3 11895-11961\3 12187-12254\3;

charset Subset37 = 11021-11091\3 12255-12322\3 11963-12037\3 12188-12254\3 11607-11676\3;

charset Subset38 = 11165-11237\3 11022-11091\3;

charset Subset39 = 11094-11164\3 11389-11456\3;

charset Subset40 = 11239-11313\3 11457-11527\3 11167-11237\3;

charset Subset41 = 11896-11961\3 12110-12186\3 11238-11313\3;

charset Subset42 = 12474-12549\3 11315-11387\3;

charset Subset43 = 11606-11676\3 11964-12037\3 11316-11387\3 11530-11604\3;

charset Subset44 = 11678-11749\3 11825-11894\3 11750-11822\3 11529-11604\3 11823-11894\3;

charset Subset45 = 12323-12395\3 12324-12395\3 11605-11676\3 11679-11749\3;

charset Subset46 = 12111-12186\3 11677-11749\3 12112-12186\3;

charset Subset47 = 11824-11894\3 12256-12322\3 11897-11961\3 12397-12473\3;

charset Subset48 = 12038-12109\3;

charset Subset49 = 12189-12254\3 12398-12473\3;

charset Subset50 = 12257-12322\3 12325-12395\3 12475-12549\3;

charset Subset51 = 12396-12473\3;

charset Subset52 = 12551-14178\3 12552-14178\3 12550-14178\3;

charset Subset53 = 14181-15066\3 14180-15066\3;

charpartition PartitionFinder = GTR+I+G:Subset1, GTR+I+G:Subset2, GTR+I+G:Subset3, GTR+I+G:Subset4, GTR+I+G:Subset5, GTR+I+G:Subset6, GTR+I+G:Subset7, GTR+I+G:Subset8, GTR+I+G:Subset9, GTR+I+G:Subset10, GTR+I+G:Subset11, GTR+I+G:Subset12, GTR+I+G:Subset13, GTR+I+G:Subset14, GTR+I+G:Subset15, GTR+I+G:Subset16, GTR+I+G:Subset17, GTR+I+G:Subset18, GTR+I+G:Subset19, GTR+I+G:Subset20, GTR+I+G:Subset21, GTR+I+G:Subset22, GTR+I+G:Subset23, GTR+I+G:Subset24, GTR+I+G:Subset25, GTR+I+G:Subset26, GTR+I+G:Subset27, GTR+I+G:Subset28, GTR+I+G:Subset29, GTR+I+G:Subset30, GTR+G:Subset31, GTR+I+G:Subset32, GTR+I+G:Subset33, GTR+I+G:Subset34, GTR+G:Subset35, GTR+G:Subset36, GTR+I+G:Subset37, GTR+G:Subset38, GTR+G:Subset39, GTR+G:Subset40, GTR+G:Subset41, GTR+G:Subset42, GTR+I+G:Subset43, GTR+I+G:Subset44, GTR+G:Subset45, GTR+G:Subset46, GTR+I+G:Subset47, GTR+G:Subset48, GTR+G:Subset49, GTR+G:Subset50, GTR+G:Subset51, GTR+I+G:Subset52, GTR+I+G:Subset53;

end;

**c. BI tree optimal model**

begin mrbayes;

charset Subset1 = 1-1024\3;

charset Subset2 = 2-1024\3;

charset Subset3 = 3-1024\3;

charset Subset4 = 1025-2558\3;

charset Subset5 = 1026-2558\3;

charset Subset6 = 4144-4936\3 1027-2558\3;

charset Subset7 = 11458-11527\3 2559-3240\3;

charset Subset8 = 8837-9990\3 2560-3240\3;

charset Subset9 = 2561-3240\3;

charset Subset10 = 3241-3444\3;

charset Subset11 = 3242-3444\3 12040-12109\3;

charset Subset12 = 5292-7051\3 3243-3444\3;

charset Subset13 = 3445-4143\3;

charset Subset14 = 3446-4143\3;

charset Subset15 = 3447-4143\3 9991-10936\3;

charset Subset16 = 4145-4936\3 11459-11527\3;

charset Subset17 = 4146-4936\3;

charset Subset18 = 11092-11164\3 11390-11456\3 4937-5290\3 11166-11237\3 11093-11164\3;

charset Subset19 = 4938-5290\3;

charset Subset20 = 4939-5290\3 8838-9990\3;

charset Subset21 = 12476-12549\3 5291-7051\3;

charset Subset22 = 5293-7051\3;

charset Subset23 = 7052-7976\3 11314-11387\3;

charset Subset24 = 7053-7976\3;

charset Subset25 = 7054-7976\3 14179-15066\3;

charset Subset26 = 9992-10936\3 7977-8267\3;

charset Subset27 = 7978-8267\3 10939-11019\3;

charset Subset28 = 11240-11313\3 7979-8267\3;

charset Subset29 = 12039-12109\3 11962-12037\3 8268-8835\3;

charset Subset30 = 8269-8835\3;

charset Subset31 = 8270-8835\3;

charset Subset32 = 8836-9990\3;

charset Subset33 = 9993-10936\3;

charset Subset34 = 10937-11019\3 11528-11604\3 11752-11822\3;

charset Subset35 = 11388-11456\3 10938-11019\3;

charset Subset36 = 11751-11822\3 11020-11091\3 11895-11961\3 12187-12254\3;

charset Subset37 = 11021-11091\3 12255-12322\3 11963-12037\3 12188-12254\3 11607-11676\3;

charset Subset38 = 11165-11237\3 11022-11091\3;

charset Subset39 = 11094-11164\3 11389-11456\3;

charset Subset40 = 11239-11313\3 11457-11527\3 11167-11237\3;

charset Subset41 = 11896-11961\3 12110-12186\3 11238-11313\3;

charset Subset42 = 12474-12549\3 11315-11387\3;

charset Subset43 = 11606-11676\3 11964-12037\3 11316-11387\3 11530-11604\3;

charset Subset44 = 11678-11749\3 11825-11894\3 11750-11822\3 11529-11604\3 11823-11894\3;

charset Subset45 = 12323-12395\3 12324-12395\3 11605-11676\3 11679-11749\3;

charset Subset46 = 12111-12186\3 11677-11749\3 12112-12186\3;

charset Subset47 = 11824-11894\3 12256-12322\3 11897-11961\3 12397-12473\3;

charset Subset48 = 12038-12109\3;

charset Subset49 = 12189-12254\3 12398-12473\3;

charset Subset50 = 12257-12322\3 12325-12395\3 12475-12549\3;

charset Subset51 = 12396-12473\3;

charset Subset52 = 12551-14178\3 12552-14178\3 12550-14178\3;

charset Subset53 = 14181-15066\3 14180-15066\3;

partition PartitionFinder = 53:Subset1, Subset2, Subset3, Subset4, Subset5, Subset6, Subset7, Subset8, Subset9, Subset10, Subset11, Subset12, Subset13, Subset14, Subset15, Subset16, Subset17, Subset18, Subset19, Subset20, Subset21, Subset22, Subset23, Subset24, Subset25, Subset26, Subset27, Subset28, Subset29, Subset30, Subset31, Subset32, Subset33, Subset34, Subset35, Subset36, Subset37, Subset38, Subset39, Subset40, Subset41, Subset42, Subset43, Subset44, Subset45, Subset46, Subset47, Subset48, Subset49, Subset50, Subset51, Subset52, Subset53;

set partition=PartitionFinder;

lset applyto=(1) nst=6 rates=invgamma;

lset applyto=(2) nst=6 rates=invgamma;

lset applyto=(3) nst=6 rates=invgamma;

lset applyto=(4) nst=6 rates=invgamma;

lset applyto=(5) nst=6 rates=invgamma;

lset applyto=(6) nst=6 rates=invgamma;

lset applyto=(7) nst=6 rates=invgamma;

lset applyto=(8) nst=6 rates=invgamma;

lset applyto=(9) nst=6 rates=invgamma;

lset applyto=(10) nst=6 rates=invgamma;

lset applyto=(11) nst=6 rates=invgamma;

lset applyto=(12) nst=6 rates=invgamma;

lset applyto=(13) nst=6 rates=invgamma;

lset applyto=(14) nst=6 rates=invgamma;

lset applyto=(15) nst=6 rates=invgamma;

lset applyto=(16) nst=6 rates=invgamma;

lset applyto=(17) nst=6 rates=invgamma;

lset applyto=(18) nst=6 rates=invgamma;

lset applyto=(19) nst=6 rates=invgamma;

lset applyto=(20) nst=6 rates=invgamma;

lset applyto=(21) nst=6 rates=invgamma;

lset applyto=(22) nst=6 rates=invgamma;

lset applyto=(23) nst=6 rates=invgamma;

lset applyto=(24) nst=6 rates=invgamma;

lset applyto=(25) nst=6 rates=invgamma;

lset applyto=(26) nst=6 rates=invgamma;

lset applyto=(27) nst=6 rates=invgamma;

lset applyto=(28) nst=6 rates=invgamma;

lset applyto=(29) nst=6 rates=invgamma;

lset applyto=(30) nst=6 rates=invgamma;

lset applyto=(31) nst=6 rates=gamma;

lset applyto=(32) nst=6 rates=invgamma;

lset applyto=(33) nst=6 rates=invgamma;

lset applyto=(34) nst=6 rates=invgamma;

lset applyto=(35) nst=6 rates=gamma;

lset applyto=(36) nst=6 rates=gamma;

lset applyto=(37) nst=6 rates=invgamma;

lset applyto=(38) nst=6 rates=gamma;

lset applyto=(39) nst=6 rates=gamma;

lset applyto=(40) nst=6 rates=gamma;

lset applyto=(41) nst=6 rates=gamma;

lset applyto=(42) nst=6 rates=gamma;

lset applyto=(43) nst=6 rates=invgamma;

lset applyto=(44) nst=6 rates=invgamma;

lset applyto=(45) nst=6 rates=gamma;

lset applyto=(46) nst=6 rates=gamma;

lset applyto=(47) nst=6 rates=invgamma;

lset applyto=(48) nst=6 rates=gamma;

lset applyto=(49) nst=6 rates=gamma;

lset applyto=(50) nst=6 rates=gamma;

lset applyto=(51) nst=6 rates=gamma;

lset applyto=(52) nst=6 rates=invgamma;

lset applyto=(53) nst=6 rates=invgamma;

mcmcp ngen= 10000000 relburnin=yes burninfrac=0.25 printfreq=1000 samplefreq=1000 nchains=4 savebrlens=yes;

mcmc;

sumt;

End;
